# Strategies Beyond 3rd EGFR‐TKI Acquired Resistance: Opportunities and Challenges

**DOI:** 10.1002/cam4.70921

**Published:** 2025-05-05

**Authors:** Xuexue Zhou, Liang Zeng, Zhe Huang, Zhaohui Ruan, Huan Yan, Chun Zou, Shidong Xu, Yongchang Zhang

**Affiliations:** ^1^ Medical College Jishou University Jishou China; ^2^ Department of Medical Oncology, Lung Cancer and Gastrointestinal Unit, Hunan Cancer Hospital/the Affiliated Cancer Hospital of Xiangya School of Medicine Central South University Changsha China; ^3^ Department of Pathology and Pathophysiology, School of Basic Medical Sciences Central South University Changsha China

**Keywords:** acquired resistance mechanisms, *EGFR* mutation, NSCLC, therapeutic strategies, third‐generation EGFR‐TKI

## Abstract

The seminal identification of epidermal growth factor receptor (*EGFR*) mutations as pivotal oncogenic drivers in non‐small cell lung cancer (NSCLC) has catalyzed the evolution of biomarker‐guided therapeutic paradigms for advanced disease. Currently, third‐generation EGFR tyrosine kinase inhibitors (EGFR‐TKI) have revolutionized first‐line treatment for advanced *EGFR*‐mutated NSCLC, yet acquired resistance remains an inevitable and formidable clinical challenge. This review systematically summarizes molecular mechanisms underlying treatment resistance, with a focus on clinical challenges associated with central nervous system (CNS) metastases. Therapeutic resistance mechanisms are categorized into *EGFR*‐dependent (on‐target) pathways, typified by acquired kinase domain mutations (e.g., C797S), and *EGFR*‐independent (off‐target) pathways, involving compensatory activation of parallel signaling effectors (e.g., MET amplification, HER2 activation) or phenotypic transformation. We further evaluated contemporary diagnostic modalities for identifying resistance drivers and appraised emerging therapeutic strategies, including fourth‐generation EGFR‐TKI, various combination therapies, and antibody‐drug conjugates (ADCs), and so forth, with emphasis on ongoing clinical trials that may transform the existing treatment paradigm. By synthesizing preclinical and clinical insights, this review aims to advance mechanistic understanding and propose therapeutic strategies to overcome acquired resistance to third‐generation EGFR‐TKI in first‐line treatment.

## 1 Introduction

Non‐small cell lung cancer (NSCLC) is one of the most prevalent malignancies worldwide and a leading cause of cancer‐related mortality [1]. In Asian populations, the majority of lung cancer patients present with advanced‐stage disease at the time of initial diagnosis [2]. Globally, epidermal growth factor receptor (*EGFR*) mutations are identified in 20%–30% of advanced NSCLC cases, with a higher prevalence of 30%–50% reported in Asian populations. The most common mutations are exon 19 deletions and the L858R substitution [3]. For patients with advanced *EGFR*‐mutant NSCLC, EGFR tyrosine kinase inhibitors (EGFR‐TKI) have become the standard first‐line treatment due to their demonstrated efficacy in improving survival outcomes and clinical benefit [4, 5].

Although first‐ and second‐generation EGFR‐TKI (e.g., gefitinib, erlotinib, afatinib) demonstrate significant clinical efficacy in patients with advanced *EGFR*‐mutant NSCLC, disease progression occurs in most patients within 10–14 months of treatment initiation [6]. The *EGFR* T790M mutation has been identified as the primary mechanism driving resistance to these therapies. To address this issue, third‐generation EGFR‐TKI were developed. Multiple phase III clinical trials have demonstrated the robust efficacy of third‐generation EGFR‐TKI in patients who have developed disease progression due to the T790M mutation following prior EGFR‐TKI therapy. Additionally, these agents significantly improve outcomes in patients with central nervous system (CNS) metastases [7, 8]. Osimertinib, a third‐generation EGFR‐TKI, has demonstrated significant superiority over first‐generation EGFR‐TKI in the phase III FLAURA trial, with median progression‐free survival (PFS) and overall survival (OS) of 17.8 and 33.1 months, respectively. Nevertheless, 60%–70% of patients develop acquired resistance within two years, highlighting the need for innovative therapeutic approaches to overcome resistance mechanisms [9, 10]. Furthermore, approximately 20% of patients with advanced NSCLC present with brain metastases (BM) at diagnosis, which are associated with worse prognoses and higher rates of EGFR‐TKI resistance compared to those without CNS involvement [11, 12]. Investigating resistance mechanisms to third‐generation EGFR‐TKI is therefore essential for developing effective subsequent therapies.

Despite partial identification of resistance mechanisms to third‐generation EGFR‐TKI, approximately 30%–50% of these mechanisms remain poorly understood [13], severely limiting treatment options for resistant patients, particularly those with *EGFR*‐independent resistance. Therefore, in‐depth exploration of EGFR‐TKI resistance mechanisms and the development of novel drugs or combination strategies represent urgent clinical priorities. This review systematically summarizes acquired resistance mechanisms to third‐generation EGFR‐TKI, detection methods, potential biomarkers, and corresponding therapeutic strategies, with a focus on CNS metastasis‐related resistance mechanisms and treatment approaches.

## 2 Research Advances in Third‐Generation EGFR‐TKI

### 2.1 Introduction to *EGFR*

**FIGURE 1:**
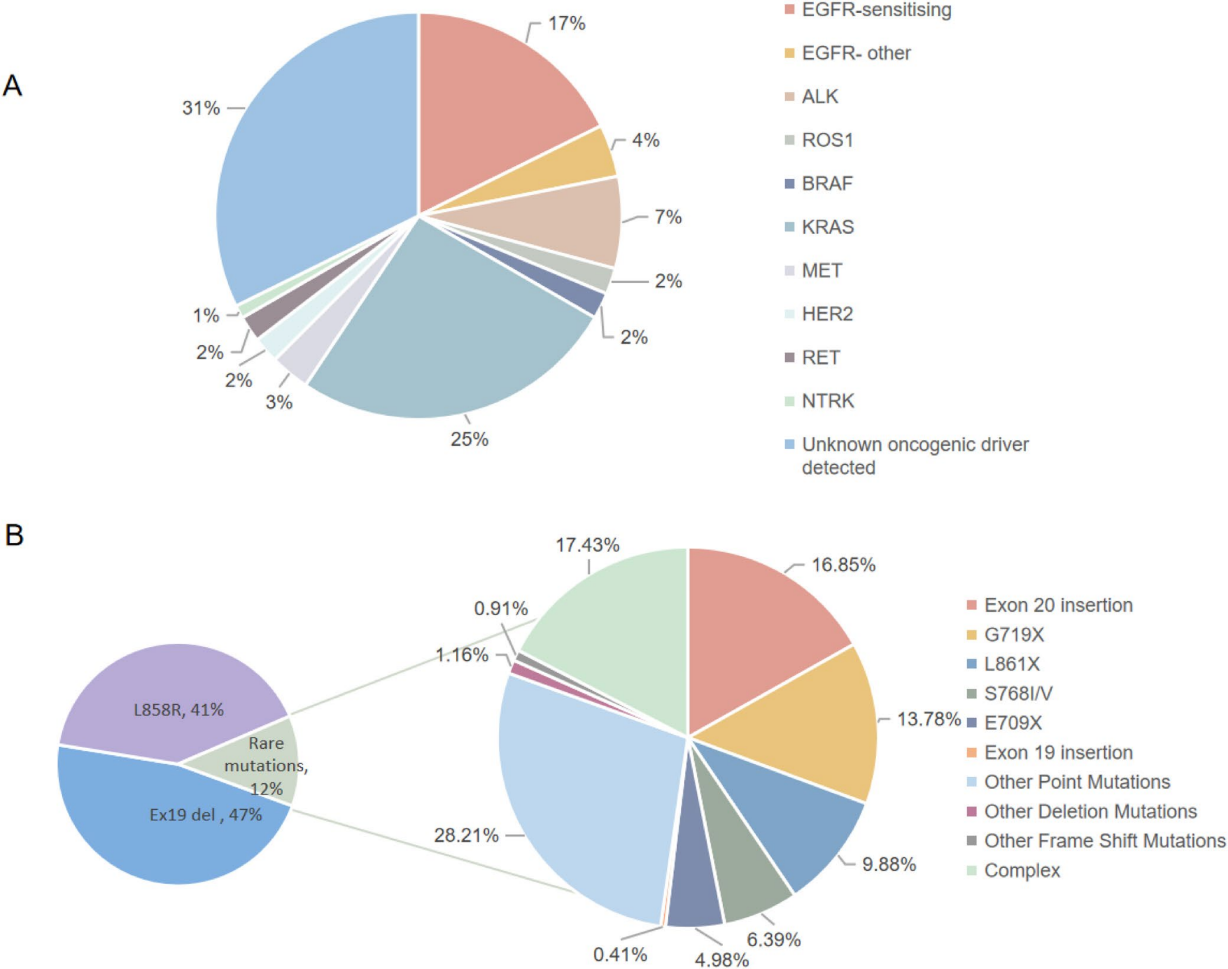
Distribution of oncoablastic drivers and *EGFR* mutations in non‐small cell lung cancer. (A) The distribution of identified oncogenic drivers among NSCLC patients. (B) Detailed breakdown of *EGFR* mutations detected among NSCLC patients.

*EGFR* (HER1/ErbB1), a member of the ErbB receptor family, consists of an extracellular ligand‐binding domain, a transmembrane helix, and an intracellular tyrosine kinase domain. Under normal physiological conditions, EGFR exists as a monomer on the cell surface. Upon mutation, *EGFR* forms dimers with constitutive activation, persisting at the plasma membrane and undergoing spontaneous dimerization even in the absence of ligand binding. This leads to the sustained activation of downstream signaling pathways that regulate cell proliferation and differentiation [14]. *EGFR* mutations are major oncogenic drivers in lung adenocarcinoma, occurring in approximately 21% of patients globally (Figure 1A) [3]. Classical mutations, including exon 19 deletions and the L858R point mutation in exon 21, account for 90% of cases. Rare mutations, such as G719X, L861Q, S768I substitutions, and exon 20 insertions, are observed in 10% of patients (Figure 1B) [15, 16].

### 2.2 Third‐Generation EGFR‐TKI

As the first third‐generation EGFR‐TKI approved by the FDA and EMA, osimertinib irreversibly inhibits EGFR‐sensitizing mutations (exon 19del/L858R) and the T790M resistance mutation through covalent binding to the ATP‐binding pocket. This mechanism is mediated by the formation of a sulfhydryl adduct with the cysteine‐797 (C797) residue in the kinase domain. Notably, osimertinib exhibits approximately 200‐fold greater potency against mutant EGFR (IC50 = 12 nM) compared to wild‐type EGFR (IC50 = approximately 650 nM), ensuring selective targeting of mutant EGFR isoforms [17, 18]. Table 1 provides a summary of key late‐stage clinical trial results for several third‐generation EGFR‐TKIs, including osimertinib, furmonertinib, and aumolertinib.

**TABLE 1:** Phase 1–3 trials of third‐generation EGFR‐TKI in *EGFR* ^+^ NSCLC.

Drug	Trial number	Phase	Sample size	Study design	Result
Osimertinib [19]	NCT02296125	Phase III	556	Osimertinib verus standard EGFR‐TKI (gefitinib)	mPFS: 18.9 verus 10.2 months; the survival rate at 18 months: 83% (95% CI: 78–87) verus 71% (95% CI: 65–76); ORR: 80% verus 76%; TEAE ≥ 3: 34% vs. 45%
Furmonertinib [20]	NCT03787992	Phase III	358	Furmonertinib (80 mg/day) verus gefitinib (250 mg/day)	Furmonertinib group: mPFS:20.8 months (95% CI: 17.8–23.5); TEAE: 20 (11%). Gefitinib group: mPFS: 11.1 months (95% CI: 9.7–12.5); TEAE: 32 (18%).
Aumolertinib [21]	NCT03849768	Phase III	429	Aumolertinib (110 mg) verus gefitinib (250 mg)	Aumolertinib group: mPFS: 19.3 months (95% CI: 17.8–20.8); ORR: 73.8%; DOR: 93.0%; TEAE ≥ 3: 36.4%. Gefitinib group: mPFS: 9.9 months (95% CI: 8.3–12.6); ORR: 72.1%; DOR: 96.7%; TEAE ≥ 3: 35.8%.
Lazertinib [22]	NCT04248829	Phase III	393	Lazertinib verus gefitinib	mPFS: 20.6 verus 9.7 months; HR: 0.45 (95% CI: 0.34–0.58); 0RR: 76% verus 76% (95% CI: 0.62–1.59); the safety profiles of both treatments were consistent with their previously reported safety profiles
Befotertinib [23]	NCT03861156	Phase II	466	Cohort A: befotertinib of 50 mg once daily; Cohort B: befotertinib of 75 to 100 mg once daily	Cohorts A (50 mg), *n* = 176; ORR: 54.0%; mPFS: 12.5 months (95% CI: 11.1–13.8); TEAE ≥ 3: 20.5%; treatment‐related serious adverse events: 11.4%. Cohorts B (50 mg), *n* = 290; ORR: 67.6%; mPFS: 16.6 months (95% CI: 15.0–not evaluable [NE]); TEAE ≥ 3: 29.3%; treatment‐related serious adverse events: 10.0%
Rezivertinib [24]	NCT03812809	Phase IIb	226	Patients received rezivertinib at 180 mg orally once daily	ORR: 64.6% (95% CI: 58.0–70.8); DCR: 89.8% (95% CI: 85.1–93); mPFS: 12.2 months (95% CI: 9.6–13.9); mOS: 23.9 months (95% CI: 20.0–not calculated); of 226 patients, 188 (83.2%) had at least one treatment‐related adverse event, whereas grade more than or equal to 3 occurred in 45 (19.9%) patients. No interstitial lung disease was reported
SH‐1028 [25]	NCT03823807	Phase II	286	Part A: dose‐verification study; *n* = 59. Part B: second‐line registration study; *n* = 227	Part A: ORR: 55.9% (95% CI: 42.4–68.8); mPFS: 12.4 months (95% CI: 8.3–20.8); mOS: 26.0 months (95% CI: 23.3–not reached). Part B: ORR: 60.4% (95% CI: 53.7–66.8); mPFS: 12.6 months (95% CI: 9.7–15.3); mOS: immature; Among the 286 patients, 44 of them experienced at least one grade 3 or higher treatment‐related adverse event
D‐0316 [26]	NCT04206072	Phase III	568	Befotertinib verus icotinib	Befotertinib group: *n* = 182; mPFS: 22.1 months (95% CI: 17.9–not estimable); TEAE ≥ 3.30%. Icotinib group: *n* = 180; mPFS:13.8 months (95% CI: 12.4–15.2); TEAE ≥ 3.8%

Abbreviations: 95% CI=confidence interval; DCR = disease control rate; mPFS = median progression‐free survival; ORR = objective response rate; TEAE = treatment‐emergent adverse event.

## 3 Mechanisms of Acquired Resistance to Third‐Generation EGFR‐TKI

**FIGURE 2:**
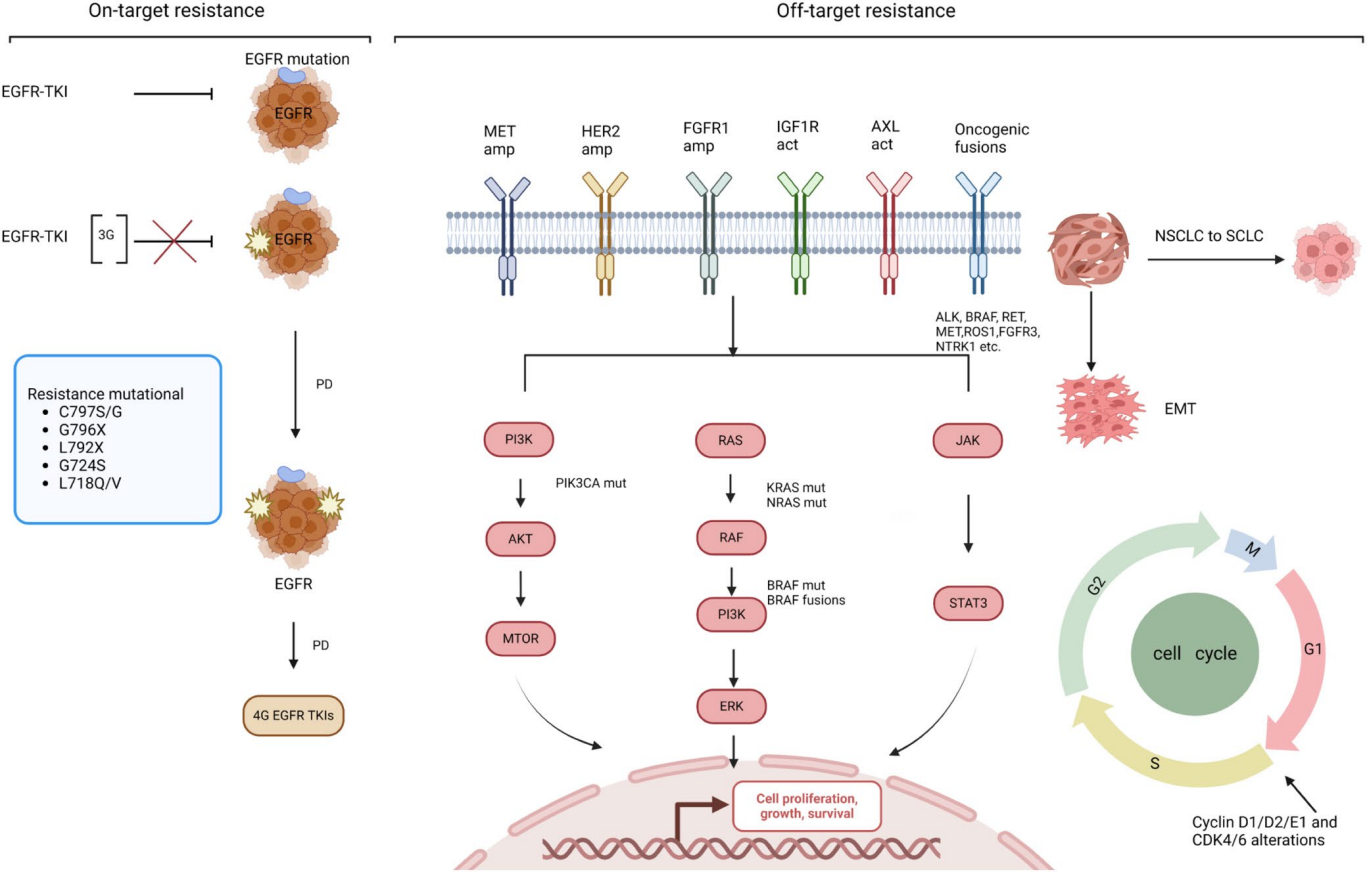
Overview of the *EGFR* signal transduction pathway model, including on‐ and off‐target mechanisms. The left panel illustrates resistance to targeted therapy due to mutations in the EGFR tyrosine kinase domain, which hinder the binding of TKIs to EGFR, thereby rendering tumor cells insensitive to EGFR inhibition. It outlines prevalent mutations following resistance to third‐generation EGFR‐TKI. Fourth‐generation EGFR‐TKI are specifically designed to target compound mutations that current FDA‐approved EGFR inhibitors fail to address. 3G, third generation; act, activation; amp, amplification; AXL, Axl receptor tyrosine kinase; EMT, epithelial–mesenchymal transition; FGFR, fibroblast growth factor receptor; IGF1R, insulin‐like growth factor 1 receptor; mut, mutation; PD, disease progression; PI3K, phosphatidylinositol‐3‐kinase.

Resistance mechanisms to third‐generation EGFR‐TKI can be broadly categorized into *EGFR*‐dependent (on‐target) and *EGFR*‐independent (off‐target) pathways. On‐target resistance primarily involves acquired mutations in the EGFR kinase domain, leading to sustained activation and signaling of receptor tyrosine kinases (RTKs). In contrast, off‐target resistance encompasses tumor cell escape from EGFR‐TKI efficacy via upregulation of downstream signaling proteins, activation of bypass signaling pathways, or phenotypic transformation. While patients treated with first‐ or second‐generation EGFR‐TKI typically develop on‐target resistance, approximately 60% is attributed to T790M mutations [27]. On‐target resistance mechanisms are observed in only 6%–17% of patients receiving third‐generation EGFR‐TKI as first‐line therapy [28, 29]. Acquired resistance mechanisms following first‐line third‐generation EGFR‐TKI treatment in *EGFR*‐mutant NSCLC are further discussed in Figure 2.

### 3.1 On‐Target Resistance

On‐target resistance often involves alterations in critical amino acid residues that disrupt TKI binding to the ATP‐binding site of the EGFR kinase domain. Currently, C797S is the most common *EGFR*‐dependent resistance mutation following third‐generation EGFR‐TKI therapy (incidence: 3.2% post‐first‐line osimertinib) [30]. This mutation involves a cysteine‐to‐serine substitution in exon 20 of the *EGFR* gene, impairing the ability of third‐generation EGFR‐TKI to form covalent bonds in the ATP‐binding pocket, thereby compromising their efficacy in suppressing EGFR activation. Notably, the C797S mutation may occur alone or coexist with the T790M mutation. When the C797S mutation and the T790M mutation are on the same allele, it is termed a cis mutation; if on different alleles, a trans mutation. This phenomenon may relate to tumor heterogeneity, selective pressure, and clonal evolution [31]. In addition to C797S, other resistance mutations include: G724S mutation located in the P‐loop region, which impedes drug binding by altering the conformation of the glycine‐rich loop [32]. G796X mutations that inhibit osimertinib binding to the EGFR kinase domain [33, 34]; L718Q/L792H mutations that induce steric hindrance or disrupt hydrogen bonding, altering kinase domain conformation [35]. L792X mutations that affect the kinase domain “hinge region,” impairing drug binding and diminishing inhibitory efficacy [33, 36]. Besides, wild‐type *EGFR* amplification (in 10%–20% of cases) can also lead to resistance by reactivating signaling pathways through heterodimerization [37].

### 3.2 Off‐Target Resistance

During treatment with third‐generation EGFR‐TKI, tumors can acquire resistance through non‐*EGFR* mutations or alterations in signaling pathways, primarily including MET amplification, gene fusions, reactivation of *EGFR* downstream signaling pathways, and histologic transformation.

#### 3.2.1 MET Amplification

**FIGURE 3:**
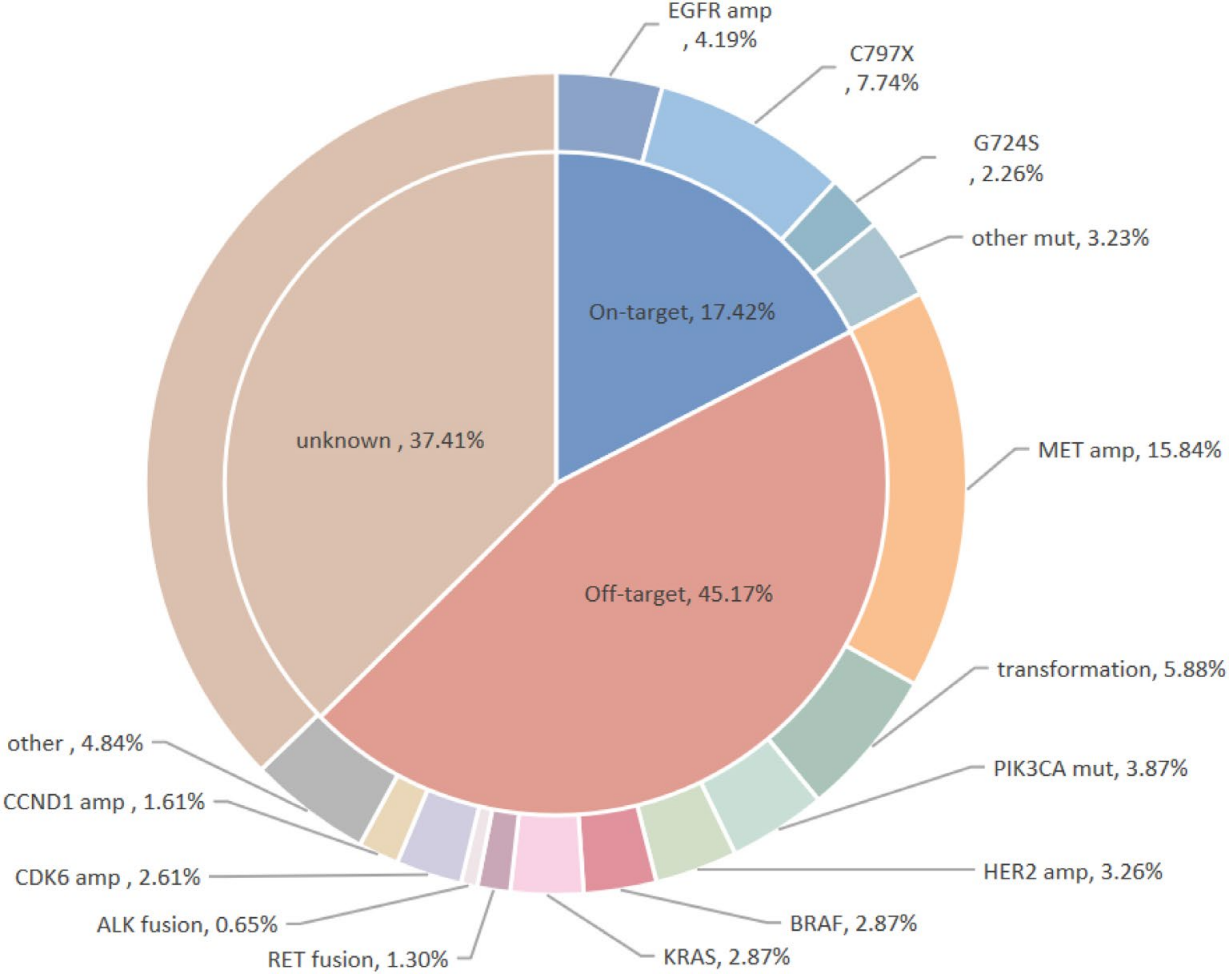
The frequency of acquired resistance mutations after first‐line treatment with third generation EGFR‐TKI. Mechanisms of resistance to first‐line osimertinib: Amp, amplification; other, CCND2, CCND3, CCNE1, CDK4, CDN2a, TERT, RB1loss, and PTEN loss; other mut, G796S, L718Q, EX20ins, and S768I; X, C797S or C797G. Acquired changes in the sample following osimertinib in one or more of the patients [31, 38, 39, 40, 41, 42].

MET amplification is currently recognized as the most common *EGFR*‐independent resistance mechanism to third‐generation EGFR‐TKI. Following first‐line osimertinib treatment, MET amplification is observed in 15.84% of patients (Figure 3). The MET proto‐oncogene, located on the long arm of chromosome 7 (7q31), encodes the MET (c‐MET) protein, a transmembrane tyrosine kinase receptor. Binding of MET to its ligand, hepatocyte growth factor (HGF) secreted by stromal cells, induces receptor dimerization and activation, thereby triggering *EGFR*‐independent downstream signaling pathways such as PI3K/AKT, JAK/STAT, and RAS/MAPK/ERK. These pathways promote resistance to third‐generation EGFR‐TKI [43]. Consequently, dual inhibition of EGFR and MET is necessary to overcome resistance driven by MET amplification. Preclinical studies have shown that combining osimertinib with MET knockdown or small‐molecule inhibitors (e.g., crizotinib) effectively reverses MET amplification–mediated resistance [44].

#### 3.2.2 Alterations in Other Tyrosine Kinase Receptors

In addition to MET amplification, aberrant activation of other tyrosine kinase receptors, such as HER2, AXL, FGFR1, and IGF1R, is closely associated with EGFR‐TKI resistance. In the AURA3 clinical trial, HER2 amplification was identified as a mechanism of acquired resistance to osimertinib, occurring in approximately 5% of patients [28]. The HER2 gene encodes the ERBB2 receptor tyrosine kinase, which predominantly exists in a monomeric state or forms heterodimers with other receptors (e.g., EGFR) in the absence of a specific ligand required for homodimerization [45]. HER2 drives resistance to third‐generation EGFR‐TKI by activating the MAPK and PI3K signaling pathways independent of EGFR activity.

AXL, another receptor tyrosine kinase, plays a significant role in tumor growth, invasion, and metastasis. Taniguchi et al. demonstrated that AXL overexpression is more pronounced in *EGFR*‐mutant lung adenocarcinoma (LUAD) compared to wild‐type *EGFR* [46]. Activated AXL promotes tumor cell survival and mediates resistance to osimertinib through interactions with EGFR and HER3. Additional mechanisms contributing to resistance include FGFR1 amplification and IGF1R activation [47, 48].

#### 3.2.3 Oncogenic Fusions

Oncogenic fusions sustain signaling pathway activation by affecting tyrosine kinases, chromatin regulators, or transcription factors. These alterations account for 1%–10% of resistance cases in NSCLC [49], involving RET, BRAF, ALK, ROS1, FGFR3, and NTRK1 [50, 51, 52]. A study of 62 patients receiving first‐line osimertinib reported that 19% exhibited off‐target resistance driven by oncogenic fusions, which were associated with poor prognosis [38].

#### 3.2.4 Reactivation of Downstream Signaling Pathways of *EGFR*

*EGFR* mediates its biological functions by activating several downstream signaling pathways, including RAS/RAF/MAPK, PI3K/AKT, and JAK/STAT, through ligand binding (observed in 10%–15% of cases) [39]. Mutations in RAS/RAF can reactivate the MAPK pathway, leading to resistance to third‐generation EGFR‐TKI [53, 54]. Additionally, ERK upregulation within the MAPK pathway has been shown to drive resistance, whereas MEK/ERK inhibitors can restore sensitivity to EGFR‐TKI [55].

Studies suggest that PIK3CA mutations and PTEN loss are key drivers of abnormal PI3K pathway activation [13], which leads to persistent activation of the PI3K/AKT/mTOR pathway. This dysregulation plays a crucial role in tumorigenesis, proliferation, migration, invasion, and therapeutic resistance. PIK3CA mutations are notably prevalent in advanced NSCLC patients treated with osimertinib [33, 56]. Wu et al. demonstrated that osimertinib increases Rab GTPase (RAB17) expression during first‐line treatment in NSCLC, promoting exosome release. These exosomes contain wild‐type EGFR proteins, activating downstream PI3K/AKT and MAPK pathways and driving osimertinib resistance [57].

Similarly, in patients resistant to third‐generation EGFR‐TKI, Liu et al. reported that STAT3 phosphorylation and dimerization enhance the expression of downstream target genes, such as Bcl‐2, MMP‐2, and VEGF, through JAK/STAT pathway activation. This mechanism bypasses *EGFR* signaling, promoting tumor cell proliferation, invasion, and anti‐apoptotic activity [58]. Furthermore, JAK/STAT activation upregulates PD‐L1 expression, enabling immune evasion and reducing EGFR‐TKI efficacy [59].

#### 3.2.5 Cell Cycle Gene Alterations

Genetic changes in the cell cycle include alterations in cyclin D1, D2, and E1 genes, CDK4 and CDK6 genes, and the CDKN2A gene [56]. Upregulation of these pro‐oncogenic genes has been observed in osimertinib‐treated patients and correlates with worse clinical outcomes [56, 60]. Research suggests that the CDK4/6‐RB signaling axis reduces EGFR‐TKI efficacy in *EGFR*‐mutant NSCLC, highlighting CDK4/6 as pivotal mediators of resistance. Thus, targeting CDK4/6 may enhance EGFR‐TKI antitumor effects.

#### 3.2.6 Histological Transformation

NSCLC can transform into small cell lung cancer (SCLC)following third‐generation EGFR‐TKI treatment failure, as shown by extensive evidence [61, 62]. This transformation involves substantial morphological and biological changes. While the mechanisms are not fully understood, these tumors retain *EGFR* mutations but lose *EGFR*‐driven oncogenic activity, rendering EGFR‐TKI ineffective. Bi‐allelic inactivation of TP53 and RB1 genes is observed in most cases of SCLC transformation [63]. Additional genetic alterations include TERT amplification, PIK3CA oncogenic mutation [64], ASCL1 transcription factor activation, and MYC activation [65, 66], which may serve as therapeutic targets for this subtype [67].

#### 3.2.7 EMT

Epithelial mesenchymal transition (EMT) is strongly associated with resistance to third‐generation EGFR‐TKI. PIM1 overexpression enhances proliferation, invasion, and drug resistance in osimertinib‐resistant cells by phosphorylating and inhibiting GSK3β, which stabilizes SNAIL and SLUG proteins. This process worsens EMT and resistance, while PIM1 inhibitors can suppress EMT and restore osimertinib sensitivity [68]. Furthermore, osimertinib‐induced TGFβ2 elevation is linked to EMT via SMAD2 activation and the NF‐κB pathway, offering important insights into resistance mechanisms [69]. Epigenomic and CRISPR/Cas9 screening highlighted the MIR141/MIR200C‐ZEB1/ZEB2‐FGFR1 axis as a central driver of EMT‐related EGFR‐TKI resistance, presenting novel diagnostic and therapeutic opportunities [70].

### 3.3 Mechanisms of Resistance to Third‐Generation EGFR‐TKI in Brain Metastasis

FLAURA2 [71], AENEAS [21], and FURLONG [20] trials demonstrated significant CNS PFS benefits of third‐generation EGFR‐TKI in *EGFR*‐mutant NSCLC with brain metastases (CNS PFS: osimertinib 18.9 months vs. first‐ /second‐generation EGFR‐TKI 10.2 months; aumolertinib 15.3 months vs. gefitinib 8.2 months; furmonertinib 20.8 months vs. gefitinib 9.8 months). However, limited blood–brain barrier penetration results in inadequate intracranial drug exposure. Additionally, molecular heterogeneity in brain metastases and the lack of prospective studies focusing on CNS‐specific resistance mechanisms present challenges in accurately understanding resistance patterns and devising therapeutic strategies.

Fu et al. found that osimertinib treatment in *EGFR*‐mutant lung cancer patients with brain metastases stimulates the release of immunogenic molecules (e.g., HMGB1, CALR), recruits T cells, enhances T‐cell infiltration, and upregulates CTLA4 expression, thereby remodeling the tumor immune microenvironment [72]. Other studies highlighted the activation of mitochondrial metabolism, particularly oxidative phosphorylation (OXPHOS), in brain metastases. Gamitrinib, an OXPHOS inhibitor, was shown to induce apoptosis and suppress tumor proliferation effectively. Combining OXPHOS inhibitors with anti‐PD‐1 immunotherapy significantly prolonged survival in preclinical models of lung cancer brain metastasis [73]. Furthermore, the RhoA/SRF pathway plays a vital role in brain metastases and osimertinib resistance. Astrocyte‐derived IL‐11 mediates immune evasion, and targeting the IL‐11/gp130/EGFR axis via RhoA inhibition holds therapeutic potential [74, 75].

Ruan et al., through single‐cell sequencing of cerebrospinal fluid (CSF) from leptomeningeal metastasis (LM) patients, identified M2‐polarized macrophages and regulatory T cells that signify an immunosuppressive microenvironment [76]. Furthermore, malignant epithelial cell clusters in the CSF of LM patients were also identified as key drivers of osimertinib resistance. These cells facilitate immune evasion through CD47‐SIRPA interactions and RNASE1_M signaling [77].

CNS progression remains a significant obstacle in NSCLC management. The findings above shed light on potential mechanisms underlying brain metastases and present therapeutic targets for further investigation to develop personalized strategies.

## 4 Liquid Biopsy and Molecular Biomarkers in EGFR‐TKI Resistance Monitoring

For patients developing resistance after third‐generation EGFR‐TKI therapy, re‐biopsy of tumor tissue is often conducted to identify resistance mechanisms and guide subsequent treatment. However, challenges associated with tissue biopsies, such as risks of invasive procedures, tumor heterogeneity, insufficient sample size, limited patient compliance, and ethical issues, can impact their feasibility. Next‐generation sequencing (NGS) has become a crucial tool for detecting resistance‐related mutations. Although plasma‐based NGS demonstrates high specificity, its sensitivity varies across platforms due to limited tumor‐derived DNA. In the BioCAST/IFCT‐1002 lung cancer study, plasma‐based NGS showed a sensitivity of 58% and a specificity of 87% [78].

In clinical settings, liquid biopsy represents a non‐invasive alternative for monitoring resistance. By analyzing circulating tumor DNA (ctDNA) in blood, liquid biopsy enables real‐time tracking of tumor‐specific genetic changes [79]. A meta‐analysis by Chen et al. revealed sensitivity and specificity rates of 69% (95% CI: 0.63–0.74) and 99% (95% CI: 0.97–1.00), respectively, for ctDNA in detecting mutations [80]. A study involving 122 NSCLC patients treated with EGFR‐TKI found that 41.8% exhibited multiple resistance mutations. Notably, hypermethylation of homeobox (HOX) genes, known regulators of tumor differentiation, was highlighted as a signature mechanism of EGFR‐TKI resistance [81].

Additionally, cell‐free DNA (cfDNA) analysis also detects small‐cell transformation in *EGFR*‐mutant adenocarcinoma, though the sensitivity for resistance mutations remains suboptimal [82]. Meanwhile, extracellular vesicle (EV) mRNA sequencing provides detailed insights into somatic mutations, resistance mechanisms, and tumor recurrence [83]. For patients with brain metastases, the limited blood–brain barrier penetration of ctDNA detection is a significant limitation, as only a small fraction of mutations are detectable in plasma. However, CSF demonstrates superiority for detecting CNS‐derived ctDNA, offering higher allele detection rates and variant allele frequencies compared to plasma, with sensitivities of 81.5% in CSF versus 62.5% in plasma [75].

Beyond liquid biopsy technologies, dysregulation of resistance‐related molecular biomarkers also provides critical insights for monitoring resistance and prognostic stratification. Research shows that PrP^C^ levels increase during EGFR‐TKI resistance, and reducing PrP^C^ expression restores sensitivity to osimertinib. Thus, PrP^C^ is recognized as a key driver of EMT‐dependent EGFR‐TKI resistance and a potential therapeutic target for *EGFR*‐mutant NSCLC patients with TKI failure [84]. Similarly, elevated serum levels of soluble cadherin‐3 (sCDH3) during EGFR‐TKI resistance correlate negatively with PFS and OS. sCDH3 enhances tumor cell invasiveness by regulating EMT, serving as a dual‐function biomarker for early resistance warning and survival prediction [85]. Additionally, TP53 co‐mutations in *EGFR*‐mutant NSCLC accelerate TKI resistance. Enrichment of APOBEC3A signatures in tumor samples from relapsed patients points to potential predictive biomarkers [86, 87]. Notably, mutations in *EGFR*, *TP53, CDKN2A, MYC, and CDKN2B* are more frequently detected in CSF ctDNA than in lung adenocarcinoma tissues (*p* < 0.05) [88]. These molecular dysregulations not only provide resistance monitoring targets but also underpin therapeutic strategies to overcome resistance.

## 5 Strategies for Overcoming Resistance to Third Generation EGFR‐TKI

### 5.1 Treatment Strategies Targeting On‐Target Resistance Mechanisms

#### 5.1.1 Targeted Rechallenge

Targeted rechallenge involves the re‐administration of targeted therapies to patients who previously discontinued treatment due to resistance or toxicity, aiming to regain clinical benefit. In cases of acquired resistance to third‐generation EGFR‐TKI, secondary mutations in the EGFR kinase domain (e.g., L718Q, G724S) retain sensitivity to first‐ or second‐generation EGFR‐TKI, likely due to allosteric effects or conformational changes in the ATP‐binding pocket. For instance, a study reported that 8 patients with osimertinib‐resistant Ex19del/G724S mutations achieved a 100% disease control rate and a median PFS of 4.5 months after rechallenge with the second‐generation TKI afatinib, significantly outperforming 15 patients treated with non‐afatinib therapies (median PFS: 1.7 months) [89]. For patients harboring the L718Q mutation, afatinib combined with an anti‐EGFR monoclonal antibody (e.g., cetuximab) has demonstrated efficacy as a salvage regimen [90]. Similarly, durable responses have been observed in patients with L718V mutations treated with afatinib or brigatinib (an EGFR/ALK dual‐target TKI) [91, 92]. A case report further highlighted sensitivity to gefitinib in a patient with the G796S mutation following disease progression on osimertinib [93].

For resistance driven by *EGFR* T790M/C797S compound mutations, therapeutic strategies are contingent on the allelic configuration. When C797S and T790M are in trans, sensitivity to combined first‐ and third‐generation EGFR‐TKI is retained [94]. In contrast, cis configurations render EGFR‐TKI ineffective, posing a significant therapeutic challenge [29]. A retrospective study of patients with cis C797S/T790M mutations showed that brigatinib combined with cetuximab achieved an objective response rate (ORR) of 60% and a median PFS of 14 months, compared to 10% ORR and 3 months median PFS in chemotherapy‐treated patients [95]. Additional strategies, such as the combination of brigatinib with chemotherapy or anti‐angiogenic agents, or osimertinib with anlotinib, have shown potential benefits for patients with cis T790M/C797S mutations [96, 97]. Despite increasing attention to cis C797S‐mediated resistance, no standardized treatment has been established, highlighting the need for prospective trials to validate safety and efficacy in this population.

#### 5.1.2 Novel Later‐Generation of EGFR Inhibitors

No EGFR‐targeted therapies are currently approved following progression on third‐generation EGFR‐TKI. However, fourth‐generation EGFR‐TKI designed to address C797S‐mediated resistance have shown encouraging potential. EAI045, which binds to a c‐helix displacement site in the kinase inactive conformation [98], exhibits preclinical efficacy particularly when combined with cetuximab [99, 100]. BBT‐176, a C797S‐selective inhibitor with broad activity against wild‐type *EGFR*, demonstrated tumor shrinkage and radiographic improvement in a Phase I trial (NCT04820023) [101]. Similarly, JBJ‐09‐063, a novel EGFR variant inhibitor, has shown significant activity against *EGFR* L858R/T790M/C797S triple mutations in preclinical models, especially when used in combination with third‐generation TKIs [102]. Other investigational C797S‐specific inhibitors include OBX02‐011 [103], BI‐4732 [104], CH7233163 [105], and JBJ‐04‐125‐02 [106]. Notably, BLU‐945, a targeted agent designed for triple‐resistant *EGFR* mutations (*EGFR* 19del/L858R/T790M/C797S), showed tumor reduction in Phase I/II trials as monotherapy or in combination with osimertinib, but its high‐dose cohort was discontinued due to hepatic toxicity [107]. As with third‐generation EGFR‐TKI, novel agents targeting the *EGFR* C797S resistance mutation may ultimately encounter resistance challenges. Optimally, fourth‐generation EGFR‐TKI should not only overcome C797S‐mediated resistance but also demonstrate improved efficacy in patients with CNS metastases. Currently, several fourth‐generation EGFR‐TKI have entered clinical trials, providing new options to combat resistance to third‐generation therapies.

#### 5.1.3 Targeted Protein Degraders

PROTACs (Proteolysis Targeting Chimeras) represent a novel class of tumor‐targeting drugs that promote the ubiquitination and degradation of target proteins (e.g., EGFR) by recruiting E3 ubiquitin ligases, thereby addressing the limitations of conventional TKIs, which only inhibit kinase activity [108]. HJM‐561 and PROTAC‐12 are orally administered PROTACs designed to target the *EGFR* L858R/T790M/C797S triple mutation (including cis‐C797S); notably, HJM‐561 achieved an 82% reduction in tumor volume without recurrence in a PDX model of *EGFR*‐mutated NSCLC [109, 110]. Currently, multiple PROTACs, such as HSK40118 (NCT06050980) and CFT8919 (NCT06641609), are in phase I clinical trials involving patients with *EGFR*‐mutant NSCLC. While PROTACs offer a promising therapeutic approach for *EGFR*‐mutant lung cancer, particularly in patients with multidrug resistance, they still face critical challenges, including issues related to drug delivery, toxicity, and evaluating their compatibility with combination therapies.

### 5.2 Treatment Strategies Targeting Off‐Target Resistance Mechanisms

#### 5.2.1 Combination of EGFR‐TKI With Signal Pathway‐Related Inhibitors

**FIGURE 4:**
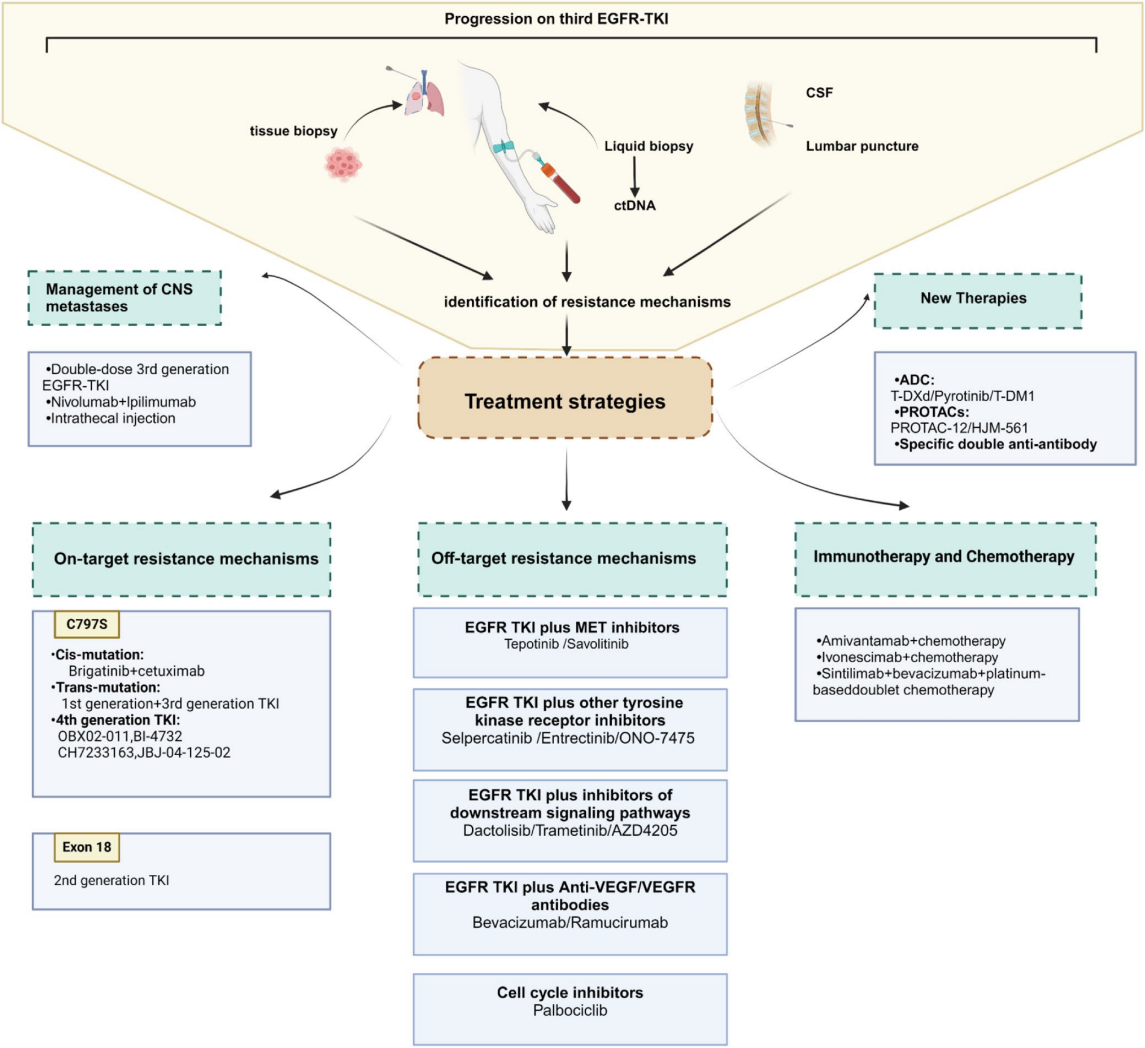
Potential therapeutic strategies targeting resistance mechanisms to third‐generation EGFR‐TKI. For patients experiencing disease progression following third‐generation EGFR‐TKI therapy, molecular profiling should be performed through tissue biopsy combined with liquid biopsy (e.g., ctDNA analysis) to dynamically analyze the genomic evolution of tumors, clarify resistance mechanisms, and guide subsequent precision treatment. This figure delineates stratified intervention strategies post‐resistance to third‐generation EGFR‐TKI, including targeting specific mutations, bypass activation, aberrant downstream signaling pathways, and novel drug technologies. ADC, antibody‐drug conjugate; CNS, central nervous system; CSF, cerebrospinal fluid; ctDNA, circulating tumor DNA; PROTAC, Proteolysis‐Targeting Chimera; T‐DM1, trastuzumab emtansine; T‐DXd, trastuzumab deruxtecan; VEGF/VEGFR, vascular endothelial growth factor/receptor.

To address off‐target resistance following first‐line treatment with third‐generation EGFR‐TKI, a promising therapeutic approach is combining EGFR‐TKI with agents targeting activated bypass signaling pathways. These combinations include, but are not limited to, EGFR‐TKI plus MET inhibitors, other receptor tyrosine kinase inhibitors, anti‐angiogenic agents, or cell cycle inhibitors (Figure 4).

##### 5.2.1.1 EGFR TKI Plus MET Tyrosine Kinase Receptor Inhibitors

Emerging clinical evidence indicates that combining osimertinib with MET inhibitors can effectively overcome MET amplification‐mediated resistance. Case reports have shown that the combination of osimertinib and crizotinib achieves a median PFS ranging from 3 to 19 months [111, 112]. In the TATTON study, the osimertinib plus savolitinib 600 mg cohort demonstrated an ORR of 33%–67%, while the 300 mg cohort showed an ORR of 62%, with median PFS of 5.5–11.1 months and 9.0 months, respectively [113]. Similarly, the INSIGHT trial revealed that combining gefitinib with tepotinib significantly improved PFS and OS compared to chemotherapy, achieving an ORR of 66.7% versus 42.9%, with median PFS of 4.9 months versus 4.4 months [114, 115]. Furthermore, the EGFR‐MET bispecific antibody amivantamab has emerged as a novel therapeutic option for patients with MET alterations. Table 2 highlights ongoing phase I/II clinical trials targeting MET amplification.

**TABLE 2:** Current ongoing early phase I/II trials of various MET amplification inhibitors.

Drug name	Disease	Clinical trial (phase)	*N*	Study design	Result
Savolitinib [113]	EGFR and MET amplificationin NSCLC	NCT02143466 (Ib)	180	Savolitinib + osimertinib	Part B: savolitinib 600 mg once daily (q.d.) + osimertinib 80 mg q.d; ORR: 33%–67%; mPFS:5.5–11.1 month. Part D: 300 mg once daily (q.d.) + osimertinib 80 mg q.d; ORR: 62%; mPFS: 9.0 months
Tepotinib [116]	EGFR and MET amplificationin NSCLC	INSIGHT (II)	55	Tepotinib + gefitinib versus chemotherapy	mPFS: 4.9 versus 4.4 months; (stratified HR, 0.67; 90% CI: 0.35–1.28)
Tepotinib [117]	EGFR and MET amplificationin NSCLC	NCT03940703 (II)	128	Tepotinib + osimertinib	ORR: 50.0% (95% CI: 39.7–60.3; 49 of 98 patients); the most common treatment‐related grade 3 or worse adverse events were peripheral oedema (six [5%] of 128 patients)

Abbreviations: 95% CI = confidence interval; mPFS = median progression‐free survival; ORR = objective response rate.

##### 5.2.1.2 EGFR TKI Plus Other Tyrosine Kinase Receptor Inhibitors

Targeted therapies have been developed for various oncogenic fusions, with promising advancements in overcoming resistance mechanisms. In the prospective clinical trial NCT03906331, the combination of osimertinib and selpercatinib demonstrated safety and efficacy in NSCLC patients with acquired RET fusions, suggesting its therapeutic potential [118]. A case report highlighted that combining osimertinib with BRAF kinase inhibitors (trametinib or dabrafenib) effectively overcomes MKRN1‐BRAF fusion‐mediated resistance [119, 120]. Osimertinib combined with alectinib or crizotinib effectively manages EML4‐ALK or PLEKHA7‐ALK fusions [121]. Additionally, a case study reported clinical benefit from the combination of aumolertinib and crizotinib in a patient with concurrent *EGFR* exon 19 deletion and SLC34A2‐ROS1 fusion [122]. Clinical studies also demonstrate that entrectinib exhibits a high overall response rate (ORR) against ROS1 fusions, and the combination of osimertinib and entrectinib may represent a novel option for advanced‐stage patients [123]. These findings indicate that combining EGFR‐TKI with fusion‐targeted inhibitors is a rational strategy to counteract resistance driven by oncogenic fusions and could enhance therapeutic outcomes.

Tyrosine kinase receptor resistance mechanisms beyond MET have been explored, with studies revealing that anti‐AXL therapies, such as monoclonal antibody mAb654 or receptor inhibitors like bemcentinib, display efficacy when combined with osimertinib and cetuximab. Triple therapy incorporating osimertinib, cetuximab, and an anti‐AXL agent demonstrated preliminary efficacy, leading to the development of bispecific antibodies targeting both AXL and EGFR. Clinical data suggest that these bispecific antibodies combined with osimertinib can sustainably suppress tumor recurrence, although further validation is needed [124]. However, combining osimertinib with ONO‐7475 (an AXL inhibitor) has been shown to upregulate FGF2 via the c‐Myc pathway, thereby activating FGFR1. FGFR1 bypasses EGFR signaling inhibition by activating the PI3K/AKT pathway, but its inhibition enhances therapeutic efficacy. In lung cancer models, Ryota Nakamura et al. combined osimertinib, ONO‐7475, and BGJ398 (an FGFR inhibitor) and observed significantly increased tumor cell apoptosis, identifying FGFR1 as a promising therapeutic target [125]. Similarly, IGF1R activation promotes MAPK signaling, and in vitro studies demonstrated that the cIGF1R‐encoded peptide C‐IGF1R acts as a molecular switch by suppressing mitophagy in resistant persister tumor cells and facilitating apoptosis. Additionally, cIGF1R enhances EGFR‐TKI antitumor activity by inhibiting IGF1R precursor gene splicing, offering novel avenues to address EGFR‐TKI resistance [126]. Currently, dual kinase inhibitors targeting both IGF1R and EGFR have been developed [127].

##### 5.2.1.3 EGFR TKI Plus Inhibitors of Downstream Signaling Pathways

*EGFR* activates downstream signaling pathways, including PI3K, MAPK, and JAK/STAT. Inhibiting the reactivation of these pathways is an effective strategy to overcome EGFR‐TKI resistance. Studies have demonstrated that combining the PI3K/mTOR inhibitor dactolisib with osimertinib effectively overcomes resistance in both in vitro and in vivo models [128]. Similarly, co‐treatment with the MEK inhibitor trametinib and osimertinib restores sensitivity to osimertinib [129]. Histone deacetylase (HDAC) inhibitors reduce EGFR expression levels and downregulate EGFR‐induced phosphorylation of AKT and ERK [130]. Based on these findings, the combination of the HDAC inhibitor vorinostat and the ALK inhibitor brigatinib significantly enhances antitumor effects against *EGFR* L858R/T790M/C797S‐mutated lung adenocarcinoma cells. Concurrently, therapeutic strategies combining MEK inhibitors targeting RAS‐MAPK pathway reactivation with SHP2 inhibitors (e.g., RMC‐4630, JAB‐3312, SHP099) against RTK‐driven resistance are under active investigation (Table 3). In the JAK/STAT pathway, JAK1 is a key driver of STAT3 phosphorylation and signal transduction, making selective JAK1 inhibition a promising approach to address resistance. The JAK1 kinase inhibitor AZD4205 synergizes with osimertinib to enhance antitumor activity in NSCLC xenograft models [131]. Additionally, a clinical trial evaluating the combination of osimertinib and another JAK1 inhibitor, itacitinib, is ongoing (NCT02917993).

**TABLE 3:** Ongoing clinical trials addressing osimertinib resistance.

Treatment	Phase	Population and biomarker	Main outcome	NCT number
On‐target				
Lazertinib + amivantamab + carboplatin + pemetrexed versus amivantamab + carboplatin + pemetrexed	III	*EGFR*m NSLSC. Progression after osimertinib treatment	PFS. OS. Safety	NCT04988295
JIN‐A02	I/II	*EGFR*m of C797S or T790M	MTD. RP2D. Safety	NCT05394831
APG‐1252 + Osimertinib	Ib	*EGFR*m NSLSC. Progression after osimertinib treatment	MTD. RP2D. Safety	NCT04001777
Different doses of sulfamethoxazole furmonertinib	II	*EGFR*m NSLSC. Progression after osimertinib treatment	MTD. Safety	NCT06394674
Off‐target				
Osimertinib + abemaciclib	II	*EGFR*m NSLSC. Progression after osimertinib treatment. Cell cycle	ORR. PFS. OS	NCT04545710
Osimertinib + dalpiciclib	II	*EGFR*m NSLSC. Progression after osimertinib treatment. Cell cycle	MTD. RP2D	NCT06363734
BPI‐1178 + osimertinib	I	*EGFR*m NSLSC. Progression after osimertinib b treatment. Cell cycle: CDK4/CDK6	ORR. PFS. Safety	NCT06362980
Osimertinib + alisertib/sapanisertib	1b	*EGFR*m NSLSC. Progression after osimertinib treatment. mTORC1/2 inhibitors	MTD. RP2D. ORR. DCR. PFS	NCT04479306
Savolitinib + osimertinib	III	*EGFR*m NSLSC. Progression after osimertinib treatment. MET amplified	ORR. PFS. Safety	NCT05261399
BBP‐398 + osimertinib	Ia/Ib	*EGFR*m NSLSC. Progression after osimertinib treatment. MET amplified	MTD. RP2D	NCT06032936
Osimertinib + savolitinib	II	*EGFR*m NSLSC. Progression after osimertinib treatment. MET amplified	RP2D. Safety	NCT03778229
Antibody drug conjugates				
Osimertinib + cetuximab + tucatinib	I	*EGFR*m NSLSC. Progression after osimertinib treatment	MTD	NCT06067776
JMT101 + osimertinib	II	*EGFR*m NSLSC. Progression after osimertinib treatment	ORR. DCR. DOR. PFS. OS	NCT06391944
HER3‐DXd (patritumab deruxtecan; U3‐1402) + osimertinib	I	*EGFR*m NSLSC. Progression after osimertinib treatment	RCD. Safety	NCT04676477
Dato‐DXd + osimertinib/Dato‐DXd/platinum‐based doublet chemotherapy	III	*EGFR*m NSLSC. Progression after osimertinib treatment.	PFS	NCT06417814
Chemotherapy and immunotherapy				
Pemetrexed/carboplatin + lazertinib	II	*EGFR*m NSLSC. Progression after osimertinib treatment	iORR. iPFS. ORR. DOR. DCR. OS	NCT05477615
Necitumumab + trastuzumab + osimertinib	Ib/II	*EGFR*m NSLSC. Progression after osimertinib treatment	RP2D. ORR	NCT04285671
Amivantamab + lazertinib + bevacizumab	II	*EGFR*m NSLSC. Progression after third generation treatment	Safety	NCT05601973
Other strategies				
Alisertib + osimertinib	I/Ib	*EGFR*m NSLSC. Progression after osimertinib treatment	MTD. Safety	NCT04085315
APG‐1252 + osimertinib	Ib	*EGFR*m NSLSC. Progression after osimertinib treatment	MTD. RP2D	NCT04001777
Quaratusugene ozeplasmid (reqorsa) + osimertinib	I/II	*EGFR*m NSLSC. Progression after osimertinib treatment	RP2D. MTD. Safety	NCT04486833

Abbreviations: CP = carboplatin and pemetrexed; DCR = disease control rate; DOR = duration of response; EGFRm = EGFR mutant; iORR = intracranial objective response rate; iPFS = intracranial progression‐free survival; MTD = maximum tolerated dose; NSCLC = non‐small cell lung cancer; ORR = objective response rate; OS=overall survival; PFS=progression‐free survival; RCD = recommended combination dose; RP2D = recommended phase 2 dose; TKI = tyrosine kinase inhibitor.

##### 5.2.1.4 EGFR‐TKI Plus Anti‐VEGF/VEGFR Antibodies

The VEGF pathway has been implicated in EGFR‐TKI resistance, functioning as a compensatory mechanism by activating alternative pathways, such as PI3K/AKT and MAPK, to sustain tumor cell survival following EGFR inhibition [132]. Currently, bevacizumab and ramucirumab are both globally approved VEGF inhibitors for advanced NSCLC. Although combining first‐generation EGFR‐TKI (e.g., erlotinib) with VEGF inhibitors has demonstrated clinical efficacy [133], the WJOG9717L and BOOSTER trials indicated that adding bevacizumab to osimertinib did not improve PFS and increased grade ≥ 3 treatment‐related adverse events [134, 135]. Encouragingly, the CTONG‐1803/ALTER‐L001 trial showed that EGFR‐TKI combined with anlotinib significantly prolonged PFS in resistant patients (9 vs. 6 months) [136], presenting a potential treatment option for specific resistance mechanisms, though further validation in larger studies is required.

##### 5.2.1.5 Cell Cycle Inhibitors

Current therapeutic strategies to target cell cycle alterations after resistance to third‐generation EGFR‐TKI focus on CDK4/6 inhibitors. Abemaciclib, a selective CDK4/6 inhibitor, has been shown to effectively suppress cell proliferation by inhibiting CDK4/6‐mediated phosphorylation of Rb, thereby arresting the cell cycle at the G1/S transition, making it a promising therapeutic option [137]. A case report demonstrated that combining osimertinib with palbociclib (a CDK4/6 inhibitor) after disease progression maintained PR for over 10 months [138]. Combining CDK4/6 inhibitors with third‐generation EGFR‐TKI may represent a novel strategy to overcome or delay resistance in this patient population [139].

#### 5.2.2 Chemotherapy and Immunotherapy

For patients with undefined resistance mechanisms (30%–50% of cases), platinum‐based doublet chemotherapy (e.g., pemetrexed–carboplatin or paclitaxel–carboplatin) remains a cornerstone, albeit with limited efficacy. While the continuation of EGFR‐TKI during chemotherapy is debated, retrospective studies suggest that combining osimertinib with pemetrexed‐platinum in progressive patients is feasible and may confer benefit [140, 141].

In resistant patients with high PD‐L1 expression (≥ 50%) and no targetable mutations, immunotherapy has potential utility. While CheckMate722 [142] and KEYNOTE‐789 [143] studies revealed that immune checkpoint inhibitors (ICIs), either as monotherapy or in combination with chemotherapy, did not significantly improve OS and highlighted the need for cautious management of immune‐related pulmonary toxicity in the *EGFR*‐mutant population. Currently, combination strategies have shown encouraging advancements. For example, the combination of sintilimab and chemotherapy achieved a median PFS of 7.0 months (vs. 4.3 months) [144]. Moreover, the PD‐1/VEGF bispecific antibody ivonescimab (AK112), combined with chemotherapy, demonstrated substantial efficacy improvements in the HARMO study [145]. Similarly, in the ongoing MARIPOSA‐2 trial, the combination of amivantamab with chemotherapy, or amivantamab‐lazertinib with chemotherapy, extended PFS (8.2/8.3 vs. 4.2 months) and intracranial PFS (12.5/12.8 vs. 8.3 months) [146], although a higher incidence of venous thromboembolism was observed (37% vs. 9%) [147]. These findings underscore the potential for immune‐chemotherapy combinations to emerge as a new standard of care for patients lacking actionable mutations following EGFR‐TKI resistance. four‐drug combination regimens are gaining attention in the post‐EGFR‐TKI resistance setting. The Phase III ORIENT‐31 study demonstrated that the combination of sintilimab, bevacizumab, pemetrexed, and cisplatin achieved a median PFS of 7.2 months compared to 4.3 months with chemotherapy alone, with an ORR of 69.5% [144]. However, the long‐term outcomes and OS data for this strategy remain under investigation, and further clinical trials are required to validate its efficacy.

For patients with small‐cell or EMT transformation following treatment with third‐generation EGFR‐TKI, platinum‐etoposide remains the standard therapy, albeit with limited progression‐free survival (PFS) benefits [148]. Preliminary studies have shown that combining chemotherapy with immunotherapy ± bevacizumab can improve outcomes (PFS: 5.1/4.1 months; OS: 20.2 vs. 7.9 months) [149]. Ongoing phase II trials (NCT05957510) are currently investigating the efficacy of chemotherapy combined with serplulimab in SCLC‐transformed NSCLC, which may provide further insights.

Currently, immunotherapy strategies have shifted from a single mode to a diversified combination mode, and immune‐combination chemotherapy with bispecific antibodies may become a new standard for patients without targeted mutations after drug resistance. Meanwhile, the exploration of four‐drug combination regimens (Platinum‐based dual chemotherapy combined with immunotherapy and anti‐angiogenic therapy (e.g., “ABCP” regimen: atezolizumab + bevacizumab + carboplatin + paclitaxel)) and novel drug combinations based on tumor heterogeneity also provides new therapeutic strategies to overcome drug resistance.

#### 5.2.3 Antibody Drug Conjugates

For patients with HER2 amplification who experience progression following third‐generation EGFR‐TKI therapy, combining EGFR‐TKI with HER2‐TKIs has demonstrated limited efficacy [150]. For instance, the pan‐HER‐TKI pyrotinib achieved a median PFS of only 6.3 months in HER2‐amplified patients [151]. Recently, antibody‐drug conjugates (ADCs) have emerged as a promising strategy to overcome HER2 amplification‐driven resistance to EGFR‐TKI due to their unique mechanism of action. ADCs utilize monoclonal antibodies to selectively target HER2 antigens on tumor cell surfaces. Upon internalization, the linker is cleaved in lysosomes, releasing a cytotoxic payload that induces tumor cell apoptosis [152].

Trastuzumab emtansine (T‐DM1), the first HER2‐targeted ADC approved for HER2‐positive metastatic breast cancer, has shown potential in addressing HER2 amplification‐driven resistance to third‐generation EGFR‐TKI. However, in previous studies, T‐DM1 demonstrated limited efficacy in heavily pretreated HER2‐overexpressing NSCLC patients (IHC 3+ cohort, *n* = 20), with an ORR of only 20% [153]. In contrast, trastuzumab deruxtecan (T‐DXd), another HER2‐targeted ADC, has shown more promising results. A Phase II study in 91 NSCLC patients reported a median PFS of 8.2 months (95% CI: 6.0–11.9) and a median OS of 17.8 months (95% CI: 13.8–22.1) [154]. Additionally, another Phase II trial evaluated T‐DXd at two dose levels (5.4 and 6.4 mg/kg) in treatment‐naïve HER2‐mutant metastatic NSCLC patients. The ORRs were 49.0% (95% CI: 39.0–59.1) and 56.0% (95% CI: 41.3–70.0), respectively, for the 5.4 and 6.4 mg/kg dose groups [155]. Beyond HER2‐targeted ADCs, agents targeting TROP2 (e.g., Dato‐DXd), HER3 (e.g., patritumab deruxtecan), cMET, and CEACAM5 are also under clinical investigation, offering new avenues for precision therapy in NSCLC [156].

### 5.3 Management of CNS Metastases

The CNS represents the most common site of distant metastasis in advanced NSCLC, with approximately 30% of patients developing BM during the disease course [11]. For *EGFR*‐mutant patients with BM, third‐generation EGFR‐TKI demonstrate significantly enhanced blood–brain barrier (BBB) penetration, achieving CNS ORR of 60%–80% in treatment‐naïve BM patients [157, 158]. The phase III FLAURA2 trial revealed CNS ORR of 73% with osimertinib combined with chemotherapy versus 69% with monotherapy [159], suggesting potential paradigm shifts in first‐line BM management through combination strategies. However, the BBB in CNS metastases limits synchronized intracranial control by systemic therapies, often resulting in insufficient drug exposure during resistance phases. Overcoming acquired CNS resistance remains a critical clinical challenge.

The BLOOM study reported significant intracranial activity of double‐dose osimertinib (160 mg/day) in *EGFR*‐mutant LM patients, achieving an ORR of 62%, median PFS of 8.6 months, and OS of 11 months [8]. Similarly, a phase II study further confirmed a median PFS of 8.0 months and OS of 13.3 months [160]. In addition, a prospective phase I/II trial (ChiCTR1800016615) showed that intrathecal pemetrexed combined with dexamethasone achieved a median OS of 9.0 months (*n* = 30; 95% CI: 6.6–11.4 months) in LM patients [161]. Ongoing trials investigating combinations such as nivolumab with ipilimumab or EGFR‐TKI paired with PARP inhibitors require further evaluation to assess safety and efficacy. Notably, Duan H et al. demonstrated that combining CTLA‐4 inhibitors with EGFR‐TKI or co‐administering PD‐1 inhibitors with oxidative phosphorylation inhibitors (e.g., gamitrinib) significantly improved survival in murine lung cancer brain metastasis models [73]. Furthermore, Adua et al. highlighted the therapeutic potential of targeting the astrocytic IL‐11/gp130/EGFR axis through RhoA inhibition [74].

Currently, clinical practice primarily relies on double‐dose TKIs and local therapies, which may temporarily mitigate CNS progression but show limited long‐term efficacy. Local treatments also carry a high risk of recurrence, while high‐dose TKIs may lead to increased treatment‐related adverse events. Future research should focus on optimizing therapeutic agents, exploring innovative combination strategies, and validating findings through large‐scale prospective studies to overcome these challenges.

## 6 Concluding and Future Perspectives

Third‐generation EGFR‐TKI, as the standard first‐line therapy for *EGFR*‐mutant advanced NSCLC, has markedly improved PFS and control of CNS metastases. However, the emergence of acquired resistance poses a significant clinical challenge. Resistance to EGFR‐TKI is classified into on‐target and off‐target mechanisms. On‐target resistance typically involves secondary mutations in the EGFR kinase domain, while off‐target resistance includes activation of bypass signaling pathways and phenotypic transformation. CNS‐specific resistance mechanisms exhibit distinctive characteristics, such as an HMGB1/CALR‐mediated immunosuppressive microenvironment and RhoA/SRF pathway activation, underscoring the need for combination treatments that address both blood–brain barrier permeability and the local tumor microenvironment. Among current strategies for monitoring EGFR‐TKI resistance, liquid biopsy approaches (e.g., ctDNA and CSF analysis) not only facilitate early identification of patients with suboptimal responses to EGFR‐TKI but also inform subsequent therapeutic decisions. However, their sensitivity is constrained by ctDNA abundance and BBB limitations, resulting in high false‐negative rates. Future advancements require the development of high‐sensitivity detection technologies (e.g., single‐cell sequencing) integrated with multi‐omics data (epigenetic and metabolomic profiles) to comprehensively elucidate resistance evolution. Emerging technologies like CRISPR/Cas9 gene editing serve as powerful tools for resistance gene screening, enabling deeper mechanistic exploration of third‐generation EGFR‐TKI resistance [162, 163].

Tailored therapeutic strategies are essential given the heterogeneity of resistance mechanisms. For on‐target resistance, fourth‐generation EGFR‐TKI (e.g., BBT‐176, JBJ‐09‐063) and proteolysis‐targeting chimeras (PROTACs) show promise against C797S mutations but require optimization for CNS penetration. Rechallenge strategies (e.g., afatinib plus cetuximab) yield transient benefits in specific mutations (e.g., G724S, L718Q), though long‐term efficacy is hindered by tumor heterogeneity. Off‐target resistance management relies on precise detection and combination targeting. For MET amplification, osimertinib plus savolitinib or amivantamab partially reverses resistance, albeit with limited durability. Targeting oncogenic fusions (e.g., RET, BRAF) via osimertinib combined with selpercatinib or dabrafenib/trametinib is feasible but requires prospective validation. CDK4/6 inhibitors (e.g., abemaciclib) enhance EGFR‐TKI sensitivity by blocking cell cycle progression, while immune‐chemotherapy combinations (e.g., sintilimab plus chemotherapy) show potential in non‐driver mutation populations, though benefits in *EGFR*‐mutant cohorts warrant cautious evaluation. ADCs offer hope for multi‐drug‐resistant patients, yet toxicity management and dynamic resistance evolution remain challenges.

Approximately 30%–50% of acquired resistance mechanisms to third‐generation EGFR‐TKI remain undefined. Future studies integrating single‐cell sequencing, spatial transcriptomics, and patient‐derived organoids are needed to decipher spatiotemporal tumor heterogeneity and resistance clonal evolution [164]. Recent studies reveal metabolic reprogramming as a pivotal resistance mechanism. Targeting PDK1 to inhibit the glycolytic checkpoint has been shown to disrupt the energy metabolic homeostasis of resistant cells [165]. Suppression of GCLC/AKR1B‐mediated glutathione biosynthesis enhances tumor cell susceptibility to oxidative damage [166, 167], while inhibition of mitochondrial oxidative phosphorylation effectively blocks metabolic compensation pathways [73]. In hypoxic niches harboring dormant tumor cells, hypoxia‐induced ERBB signaling modulator MIG6 promotes resistance through feedback activation of non‐canonical *EGFR* pathways [168]. Beyond metabolic regulation, other critical mechanisms include epigenetic modulation, genomic instability, and aberrant signaling. Notable examples encompass the USP36‐MLLT3 epigenetic loop maintaining resistance through chromatin remodeling, APOBEC3A‐mediated NF‐κB positive feedback mutagenic amplification with APOBEC3B's microenvironment‐dependent bidirectional regulation [87, 169], and the FAK‐YAP1/TEAD axis coupled with surface markers (CD70/TROP2) emerging as potential therapeutic targets against drug‐tolerant persister (DTP) cells [87]. These mechanisms provide theoretical foundations for developing strategies targeting metabolic reprogramming, epigenetic editing, and resistant stem cell eradication.

In summary, acquired resistance to third‐generation EGFR‐TKI is characterized by significant complexity and heterogeneity, emphasizing the need for precise molecular subtyping using multi‐omics analyses and dynamic monitoring tools. Combination therapies incorporating targeted agents and novel modalities (e.g., ADCs, PROTACs) represent core strategies to overcome resistance. Future research should focus on combining preclinical insights with clinical data to develop mechanism‐driven sequential regimens, potentially revolutionizing personalized management of *EGFR*‐mutant NSCLC and reshaping current therapeutic paradigms.

## Author Contributions

Conceptualization: Yongchang Zhang, Shidong Xu. Methodology: Xuexue Zhou, Liang Zeng, Zhe Huang, Zhaohui Ruan. Writing: Xuexue Zhou, Huan Yan, Chun Zou, Shidong Xu. Supervision: Shidong Xu, Liang Zeng, Yongchang Zhang. Visualization: Xuexue Zhou.

## Consent

The authors have nothing to report.

## Conflicts of Interest

All authors declared that there are no conflicts of interest. Figures were created with BioRender software (https://app.biorender.com/).

## Data Availability

The authors have nothing to report.

## References

[1] R. L. Siegel , A. N. Giaquinto , and A. Jemal , “Cancer Statistics,” CA: A Cancer Journal for Clinicians 74, no. 1 (2024): 12–49. 38230766 10.3322/caac.21820

[2] L. Zhou , X. L. Wang , Q. L. Deng , Y. Q. Du , and N. Q. Zhao , “The Efficacy and Safety of Immunotherapy in Patients With Advanced NSCLC: A Systematic Review and Meta‐Analysis,” Scientific Reports 6 (2016): 32020. 27558285 10.1038/srep32020 PMC4997317

[3] F. R. Hirsch , G. V. Scagliotti , J. L. Mulshine , et al., “Lung Cancer: Current Therapies and New Targeted Treatments,” Lancet 389, no. 10066 (2017): 299–311. 27574741 10.1016/S0140-6736(16)30958-8

[4] R. Ruiz‐Cordero and W. P. Devine , “Targeted Therapy and Checkpoint Immunotherapy in Lung Cancer,” Surgical Pathology Clinics 13, no. 1 (2020): 17–33. 32005431 10.1016/j.path.2019.11.002

[5] L. E. Hendriks , K. M. Kerr , J. Menis , et al., “Oncogene‐Addicted Metastatic Non‐Small‐Cell Lung Cancer: ESMO Clinical Practice Guideline for Diagnosis, Treatment and Follow‐Up,” Annals of Oncology 34, no. 4 (2023): 339–357. 36872130 10.1016/j.annonc.2022.12.009

[6] Q. Chen , G. Jia , X. Zhang , and W. Ma , “Targeting HER3 to Overcome EGFR TKI Resistance in NSCLC,” Frontiers in Immunology 14 (2023): 1332057. 38239350 10.3389/fimmu.2023.1332057 PMC10794487

[7] V. A. Papadimitrakopoulou , T. S. Mok , J. Y. Han , et al., “Osimertinib Versus Platinum‐Pemetrexed for Patients With EGFR T790M Advanced NSCLC and Progression on a Prior EGFR‐Tyrosine Kinase Inhibitor: AURA3 Overall Survival Analysis,” Annals of Oncology 31, no. 11 (2020): 1536–1544. 32861806 10.1016/j.annonc.2020.08.2100

[8] J. C. H. Yang , S. W. Kim , D. W. Kim , et al., “Osimertinib in Patients With Epidermal Growth Factor Receptor Mutation‐Positive Non‐Small‐Cell Lung Cancer and Leptomeningeal Metastases: The BLOOM Study,” Journal of Clinical Oncology 38, no. 6 (2020): 538–547. 31809241 10.1200/JCO.19.00457 PMC7030895

[9] S. S. Ramalingam , J. Vansteenkiste , D. Planchard , et al., “Overall Survival With Osimertinib in Untreated, EGFR‐Mutated Advanced NSCLC,” New England Journal of Medicine 382, no. 1 (2020): 41–50. 31751012 10.1056/NEJMoa1913662

[10] Y. Cheng , Y. He , W. Li , et al., “Osimertinib Versus Comparator EGFR TKI as First‐Line Treatment for EGFR‐Mutated Advanced NSCLC: FLAURA China, a Randomized Study,” Targeted Oncology 16, no. 2 (2021): 165–176, 10.1007/s11523-021-00794-6. 33544337 PMC7935816

[11] J. B. Sørensen , H. H. Hansen , M. Hansen , and P. Dombernowsky , “Brain Metastases in Adenocarcinoma of the Lung: Frequency, Risk Groups, and Prognosis,” Journal of Clinical Oncology 6, no. 9 (1988): 1474–1480. 3047337 10.1200/JCO.1988.6.9.1474

[12] D. N. Cagney , A. M. Martin , P. J. Catalano , et al., “Incidence and Prognosis of Patients With Brain Metastases at Diagnosis of Systemic Malignancy: A Population‐Based Study,” Neuro‐Oncology 19, no. 11 (2017): 1511–1521. 28444227 10.1093/neuonc/nox077 PMC5737512

[13] A. Passaro , P. A. Jänne , T. Mok , and S. Peters , “Overcoming Therapy Resistance in EGFR‐Mutant Lung Cancer,” Nature Cancer 2, no. 4 (2021): 377–391. 35122001 10.1038/s43018-021-00195-8

[14] K. M. Ferguson , M. B. Berger , J. M. Mendrola , H. S. Cho , D. J. Leahy , and M. A. Lemmon , “EGF Activates Its Receptor by Removing Interactions That Autoinhibit Ectodomain Dimerization,” Molecular Cell 11, no. 2 (2003): 507–517. 12620237 10.1016/s1097-2765(03)00047-9

[15] J. P. Robichaux , X. Le , R. S. K. Vijayan , et al., “Structure‐Based Classification Predicts Drug Response in EGFR‐Mutant NSCLC,” Nature 597, no. 7878 (2021): 732–737. 34526717 10.1038/s41586-021-03898-1 PMC8481125

[16] H. Shigematsu , L. Lin , T. Takahashi , et al., “Clinical and Biological Features Associated With Epidermal Growth Factor Receptor Gene Mutations in Lung Cancers,” Journal of the National Cancer Institute 97, no. 5 (2005): 339–346. 15741570 10.1093/jnci/dji055

[17] I. J. Z. Eide , Å. Helland , S. Ekman , et al., “Osimertinib in T790M‐Positive and ‐Negative Patients With EGFR‐Mutated Advanced Non‐Small Cell Lung Cancer (the TREM‐Study),” Lung Cancer 143 (2020): 27–35. 32200138 10.1016/j.lungcan.2020.03.009

[18] D. A. Cross , S. E. Ashton , S. Ghiorghiu , et al., “AZD9291, an Irreversible EGFR TKI, Overcomes T790M‐Mediated Resistance to EGFR Inhibitors in Lung Cancer,” Cancer Discovery 4, no. 9 (2014): 1046–1061, 10.1158/2159-8290.CD-14-0337. 24893891 PMC4315625

[19] J. C. Soria , Y. Ohe , J. Vansteenkiste , et al., “Osimertinib in Untreated EGFR‐Mutated Advanced Non‐Small‐Cell Lung Cancer,” New England Journal of Medicine 378, no. 2 (2018): 113–125, 10.1056/NEJMoa1713137. 29151359

[20] Y. Shi , G. Chen , X. Wang , et al., “Furmonertinib (AST2818) Versus Gefitinib as First‐Line Therapy for Chinese Patients With Locally Advanced or Metastatic EGFR Mutation‐Positive Non‐Small‐Cell Lung Cancer (FURLONG): A Multicentre, Double‐Blind, Randomised Phase 3 Study,” Lancet Respiratory Medicine 10, no. 11 (2022): 1019–1028. 35662408 10.1016/S2213-2600(22)00168-0

[21] S. Lu , X. Dong , H. Jian , et al., “AENEAS: A Randomized Phase III Trial of Aumolertinib Versus Gefitinib as First‐Line Therapy for Locally Advanced or Metastatic Non‐Small‐Cell Lung Cancer With EGFR Exon 19 Deletion or L858R Mutations,” Journal of Clinical Oncology 40, no. 27 (2022): 3162–3171. 35580297 10.1200/JCO.21.02641 PMC9509093

[22] B. C. Cho , M. J. Ahn , J. H. Kang , et al., “Lazertinib Versus Gefitinib as First‐Line Treatment in Patients With EGFR‐Mutated Advanced Non‐Small‐Cell Lung Cancer: Results From LASER301,” Journal of Clinical Oncology 41, no. 26 (2023): 4208–4217. 37379502 10.1200/JCO.23.00515

[23] S. Lu , Y. Zhang , G. Zhang , et al., “Efficacy and Safety of Befotertinib (D‐0316) in Patients With EGFR T790M‐Mutated NSCLC That Had Progressed After Prior EGFR Tyrosine Kinase Inhibitor Therapy: A Phase 2, Multicenter, Single‐Arm, Open‐Label Study,” Journal of Thoracic Oncology 17, no. 10 (2022): 1192–1204, 10.1016/j.jtho.2022.06.002. 35724798

[24] Y. Shi , S. Wu , K. Wang , et al., “Efficacy and Safety of Rezivertinib (BPI‐7711) in Patients With Locally Advanced or Metastatic/Recurrent EGFR T790M‐Mutated NSCLC: A Phase 2b Study,” Journal of Thoracic Oncology 17, no. 11 (2022): 1306–1317. 36049654 10.1016/j.jtho.2022.08.015

[25] A. Xiong , S. Ren , H. Liu , et al., “Efficacy and Safety of SH‐1028 in Patients With EGFR T790M‐Positive NSCLC: A Multicenter, Single‐Arm, Open‐Label, Phase 2 Trial,” Journal of Thoracic Oncology 17, no. 10 (2022): 1216–1226. 35798241 10.1016/j.jtho.2022.06.013

[26] S. Lu , J. Zhou , H. Jian , et al., “Befotertinib (D‐0316) Versus Icotinib as First‐Line Therapy for Patients With EGFR‐Mutated Locally Advanced or Metastatic Non‐Small‐Cell Lung Cancer: A Multicentre, Open‐Label, Randomised Phase 3 Study,” Lancet Respiratory Medicine 11, no. 10 (2023): 905–915. 37244266 10.1016/S2213-2600(23)00183-2

[27] D. Yu , W. Zhao , K. A. Vallega , and S. Y. Sun , “Managing Acquired Resistance to Third‐Generation EGFR Tyrosine Kinase Inhibitors Through Co‐Targeting MEK/ERK Signaling,” Lung Cancer 12 (2021): 1–10. 33574724 10.2147/LCTT.S293902 PMC7872905

[28] J. Chmielecki , T. Mok , Y. L. Wu , et al., “Analysis of Acquired Resistance Mechanisms to Osimertinib in Patients With EGFR‐Mutated Advanced Non‐Small Cell Lung Cancer From the AURA3 Trial,” Nature Communications 14, no. 1 (2023): 1071. 10.1038/s41467-023-35962-x PMC9971022 36849516

[29] K. Fu , F. Xie , F. Wang , and L. Fu , “Therapeutic Strategies for EGFR‐Mutated Non‐Small Cell Lung Cancer Patients With Osimertinib Resistance,” Journal of Hematology & Oncology 15, no. 1 (2022): 173. 36482474 10.1186/s13045-022-01391-4 PMC9733018

[30] A. Leonetti , S. Sharma , R. Minari , P. Perego , E. Giovannetti , and M. Tiseo , “Resistance Mechanisms to Osimertinib in EGFR‐Mutated Non‐Small Cell Lung Cancer,” British Journal of Cancer 121, no. 9 (2019): 725–737. 31564718 10.1038/s41416-019-0573-8 PMC6889286

[31] N. Roper , A. L. Brown , J. S. Wei , et al., “Clonal Evolution and Heterogeneity of Osimertinib Acquired Resistance Mechanisms in EGFR Mutant Lung Cancer,” Cell Reports Medicine 1, no. 1 (2020): 100007. 32483558 10.1016/j.xcrm.2020.100007 PMC7263628

[32] Y. Zhang , B. He , D. Zhou , M. Li , and C. Hu , “Newly Emergent Acquired EGFR Exon 18 G724S Mutation After Resistance of a T790M Specific EGFR Inhibitor Osimertinib in Non‐Small‐Cell Lung Cancer: A Case Report,” Oncotargets and Therapy 12 (2019): 51–56. 30588029 10.2147/OTT.S188612 PMC6302808

[33] Z. Yang , N. Yang , Q. Ou , et al., “Investigating Novel Resistance Mechanisms to Third‐Generation EGFR Tyrosine Kinase Inhibitor Osimertinib in Non‐Small Cell Lung Cancer Patients,” Clinical Cancer Research 24, no. 13 (2018): 3097–3107. 29506987 10.1158/1078-0432.CCR-17-2310

[34] S. J. Klempner , P. Mehta , A. B. Schrock , S. M. Ali , and S. I. Ou , “Cis‐Oriented Solvent‐Front EGFR G796S Mutation in Tissue and ctDNA in a Patient Progressing on Osimertinib: A Case Report and Review of the Literature,” Lung Cancer 8 (2017): 241–247. 29255376 10.2147/LCTT.S147129 PMC5723122

[35] I. A. Imam , S. Al Adawi , X. Liu , et al., “L858R/L718Q and L858R/L792H Mutations of EGFR Inducing Resistance Against Osimertinib by Forming Additional Hydrogen Bonds,” Proteins 93, no. 3 (2024): 673–683. 39494831 10.1002/prot.26761 PMC12036761

[36] K. Chen , F. Zhou , W. Shen , et al., “Novel Mutations on EGFR Leu792 Potentially Correlate to Acquired Resistance to Osimertinib in Advanced NSCLC,” Journal of Thoracic Oncology 12, no. 6 (2017): e65–e68. 28093244 10.1016/j.jtho.2016.12.024

[37] S. Nukaga , H. Yasuda , K. Tsuchihara , et al., “Amplification of EGFR Wild‐Type Alleles in Non‐Small Cell Lung Cancer Cells Confers Acquired Resistance to Mutation‐Selective EGFR Tyrosine Kinase Inhibitors,” Cancer Research 77, no. 8 (2017): 2078–2089. 28202511 10.1158/0008-5472.CAN-16-2359

[38] A. J. Schoenfeld , J. M. Chan , D. Kubota , et al., “Tumor Analyses Reveal Squamous Transformation and Off‐Target Alterations as Early Resistance Mechanisms to First‐Line Osimertinib in EGFR‐Mutant Lung Cancer,” Clinical Cancer Research 26, no. 11 (2020): 2654–2663. 31911548 10.1158/1078-0432.CCR-19-3563 PMC7448565

[39] N. J. Choudhury , A. Marra , J. S. Y. Sui , et al., “Molecular Biomarkers of Disease Outcomes and Mechanisms of Acquired Resistance to First‐Line Osimertinib in Advanced EGFR‐Mutant Lung Cancers,” Journal of Thoracic Oncology 18, no. 4 (2023): 463–475. 36494075 10.1016/j.jtho.2022.11.022 PMC10249779

[40] J. Chmielecki , J. E. Gray , Y. Cheng , et al., “Candidate Mechanisms of Acquired Resistance to First‐Line Osimertinib in EGFR‐Mutated Advanced Non‐Small Cell Lung Cancer,” Nature Communications 14, no. 1 (2023): 1070. 10.1038/s41467-023-35961-y PMC9971254 36849494

[41] A. Leonetti , M. Verzè , R. Minari , et al., “Resistance to Osimertinib in Advanced EGFR‐Mutated NSCLC: A Prospective Study of Molecular Genotyping on Tissue and Liquid Biopsies,” British Journal of Cancer 130, no. 1 (2024): 135–142. 37938348 10.1038/s41416-023-02475-9 PMC10781773

[42] J. Chen , F. Facchinetti , F. Braye , et al., “Single‐Cell DNA‐seq Depicts Clonal Evolution of Multiple Driver Alterations in Osimertinib‐Resistant Patients,” Annals of Oncology 33, no. 4 (2022): 434–444. 35066105 10.1016/j.annonc.2022.01.004

[43] Q. Wang , S. Yang , K. Wang , and S. Y. Sun , “MET Inhibitors for Targeted Therapy of EGFR TKI‐Resistant Lung Cancer,” Journal of Hematology & Oncology 12, no. 1 (2019): 63. 31227004 10.1186/s13045-019-0759-9 PMC6588884

[44] P. Shi , Y. T. Oh , G. Zhang , et al., “Met Gene Amplification and Protein Hyperactivation Is a Mechanism of Resistance to Both First and Third Generation EGFR Inhibitors in Lung Cancer Treatment,” Cancer Letters 380, no. 2 (2016): 494–504. 27450722 10.1016/j.canlet.2016.07.021

[45] S. P. Kennedy , J. F. Hastings , J. Z. Han , and D. R. Croucher , “The Under‐Appreciated Promiscuity of the Epidermal Growth Factor Receptor Family,” Frontiers in Cell and Developmental Biology 4 (2016): 88, 10.3389/fcell.2016.00088. 27597943 PMC4992703

[46] H. Taniguchi , T. Yamada , R. Wang , et al., “AXL Confers Intrinsic Resistance to Osimertinib and Advances the Emergence of Tolerant Cells,” Nature Communications 10, no. 1 (2019): 259. 10.1038/s41467-018-08074-0 PMC6335418 30651547

[47] T. M. Kim , A. Song , D. W. Kim , et al., “Mechanisms of Acquired Resistance to AZD9291: A Mutation‐Selective, Irreversible EGFR Inhibitor,” Journal of Thoracic Oncology 10, no. 12 (2015): 1736–1744, 10.1097/JTO.0000000000000688. 26473643

[48] D. Hayakawa , F. Takahashi , Y. Mitsuishi , et al., “Activation of Insulin‐Like Growth Factor‐1 Receptor Confers Acquired Resistance to Osimertinib in Non‐Small Cell Lung Cancer With EGFR T790M Mutation,” Thoracic Cancer 11, no. 1 (2020): 140–149. 31758670 10.1111/1759-7714.13255 PMC6938756

[49] A. J. Piper‐Vallillo , L. V. Sequist , and Z. Piotrowska , “Emerging Treatment Paradigms for EGFR‐Mutant Lung Cancers Progressing on Osimertinib: A Review,” Journal of Clinical Oncology 38, no. 25 (2020): 2926–2936. 10.1200/JCO.19.03123 32552277

[50] M. Offin , R. Somwar , N. Rekhtman , et al., “Acquired ALK and RET Gene Fusions as Mechanisms of Resistance to Osimertinib in EGFR‐Mutant Lung Cancers,” JCO Precision Oncology 2 (2018): 18.00126. 10.1200/PO.18.00126 PMC6447364 30957057

[51] L. Zeng , N. Yang , and Y. Zhang , “GOPC‐ROS1 Rearrangement as an Acquired Resistance Mechanism to Osimertinib and Responding to Crizotinib Combined Treatments in Lung Adenocarcinoma,” Journal of Thoracic Oncology 13, no. 7 (2018): e114–e116. 29935846 10.1016/j.jtho.2018.02.005

[52] H. Xia , X. Xue , H. Ding , et al., “Evidence of NTRK1 Fusion as Resistance Mechanism to EGFR TKI in EGFR+ NSCLC: Results From a Large‐Scale Survey of NTRK1 Fusions in Chinese Patients With Lung Cancer,” Clinical Lung Cancer 21, no. 3 (2020): 247–254. 31761448 10.1016/j.cllc.2019.09.004

[53] M. Vojnic , D. Kubota , C. Kurzatkowski , et al., “Acquired BRAF Rearrangements Induce Secondary Resistance to EGFR Therapy in EGFR‐Mutated Lung Cancers,” Journal of Thoracic Oncology 14, no. 5 (2019): 802–815. 30831205 10.1016/j.jtho.2018.12.038 PMC6486868

[54] C. A. Eberlein , D. Stetson , A. A. Markovets , et al., “Acquired Resistance to the Mutant‐Selective EGFR Inhibitor AZD9291 Is Associated With Increased Dependence on RAS Signaling in Preclinical Models,” Cancer Research 75, no. 12 (2015): 2489–2500. 25870145 10.1158/0008-5472.CAN-14-3167 PMC4605607

[55] D. Ercan , C. Xu , M. Yanagita , et al., “Reactivation of ERK Signaling Causes Resistance to EGFR Kinase Inhibitors,” Cancer Discovery 2, no. 10 (2012): 934–947. 22961667 10.1158/2159-8290.CD-12-0103 PMC3477553

[56] X. Le , S. Puri , M. V. Negrao , et al., “Landscape of EGFR‐Dependent and ‐Independent Resistance Mechanisms to Osimertinib and Continuation Therapy Beyond Progression in EGFR‐Mutant NSCLC,” Clinical Cancer Research 24, no. 24 (2018): 6195–6203. 30228210 10.1158/1078-0432.CCR-18-1542 PMC6295279

[57] S. Wu , M. Luo , K. K. W. To , et al., “Intercellular Transfer of Exosomal Wild Type EGFR Triggers Osimertinib Resistance in Non‐Small Cell Lung Cancer,” Molecular Cancer 20, no. 1 (2021): 17, 10.1186/s12943-021-01307-9. 33461557 PMC7812728

[58] Z. Liu , L. Ma , Y. Sun , W. Yu , and X. Wang , “Targeting STAT3 Signaling Overcomes Gefitinib Resistance in Non‐Small Cell Lung Cancer,” Cell Death & Disease 12, no. 6 (2021): 561. 34059647 10.1038/s41419-021-03844-z PMC8166856

[59] J. Lu , J. Li , Z. Lin , et al., “Reprogramming of TAMs via the STAT3/CD47‐SIRPα Axis Promotes Acquired Resistance to EGFR‐TKI in Lung Cancer,” Cancer Letters 564 (2023): 216205, 10.1016/j.canlet.2023.216205. 37146936

[60] J. Lee , H. S. Kim , B. Lee , et al., “Genomic Landscape of Acquired Resistance to Third‐Generation EGFR Tyrosine Kinase Inhibitors in EGFR T790M‐Mutant Non‐Small Cell Lung Cancer,” Cancer 126, no. 11 (2020): 2704–2712. 32154925 10.1002/cncr.32809

[61] X. Yin , Y. Li , H. Wang , et al., “Small Cell Lung Cancer Transformation: From Pathogenesis to Treatment,” Seminars in Cancer Biology 86, no. Pt 2 (2022): 595–606. 35276343 10.1016/j.semcancer.2022.03.006

[62] Y. Taniguchi , H. Horiuchi , T. Morikawa , and K. Usui , “Small‐Cell Carcinoma Transformation of Pulmonary Adenocarcinoma After Osimertinib Treatment: A Case Report,” Case Reports Oncology 11, no. 2 (2018): 323–329. 10.1159/000489603 PMC6006630 29928211

[63] J. K. Lee , J. Lee , S. Kim , et al., “Clonal History and Genetic Predictors of Transformation Into Small‐Cell Carcinomas From Lung Adenocarcinomas,” Journal of Clinical Oncology 35, no. 26 (2017): 3065–3074. 28498782 10.1200/JCO.2016.71.9096

[64] N. Marcoux , S. N. Gettinger , G. O'Kane , et al., “EGFR‐Mutant Adenocarcinomas That Transform to Small‐Cell Lung Cancer and Other Neuroendocrine Carcinomas: Clinical Outcomes,” Journal of Clinical Oncology 37, no. 4 (2019): 278–285, 10.1200/JCO.18.01585. 30550363 PMC7001776

[65] H. Koba , H. Kimura , T. Yoneda , et al., “NOTCH Alteration in EGFR‐Mutated Lung Adenocarcinoma Leads to Histological Small‐Cell Carcinoma Transformation Under EGFR‐TKI Treatment,” Translational Lung Cancer Research 10, no. 11 (2021): 4161–4173. 35004247 10.21037/tlcr-21-536 PMC8674607

[66] E. E. Gardner , E. M. Earlie , K. Li , et al., “Lineage‐Specific Intolerance to Oncogenic Drivers Restricts Histological Transformation,” Science 383, no. 6683 (2024): eadj1415. 38330136 10.1126/science.adj1415 PMC11155264

[67] A. Mc Leer , M. Foll , M. Brevet , et al., “Detection of Acquired TERT Amplification in Addition to Predisposing p53 and Rb Pathways Alterations in EGFR‐Mutant Lung Adenocarcinomas Transformed Into Small‐Cell Lung Cancers,” Lung Cancer 167 (2022): 98–106. 35183375 10.1016/j.lungcan.2022.01.008

[68] J. Zhou , X. Wang , Z. Li , et al., “PIM1 Kinase Promotes EMT‐Associated Osimertinib Resistance via Regulating GSK3β Signaling Pathway in EGFR‐Mutant Non‐Small Cell Lung Cancer,” Cell Death & Disease 15, no. 9 (2024): 644. 39227379 10.1038/s41419-024-07039-0 PMC11372188

[69] X. M. Jiang , Y. L. Xu , L. W. Yuan , et al., “TGFβ2‐Mediated Epithelial‐Mesenchymal Transition and NF‐κB Pathway Activation Contribute to Osimertinib Resistance,” Acta Pharmacologica Sinica 42, no. 3 (2021): 451–459. 32678313 10.1038/s41401-020-0457-8 PMC8027198

[70] J. Vad‐Nielsen , N. H. Staunstrup , M. L. Kjeldsen , et al., “Genome‐Wide Epigenetic and mRNA‐Expression Profiling Followed by CRISPR/Cas9‐Mediated Gene‐Disruptions Corroborate the MIR141/MIR200C‐ZEB1/ZEB2‐FGFR1 Axis in Acquired EMT‐Associated EGFR TKI‐Resistance in NSCLC Cells,” Translational Lung Cancer Research 12, no. 1 (2023): 42–65. 36762066 10.21037/tlcr-22-507 PMC9903082

[71] D. Planchard , P. A. Jänne , Y. Cheng , et al., “Osimertinib With or Without Chemotherapy in EGFR‐Mutated Advanced NSCLC,” New England Journal of Medicine 389, no. 21 (2023): 1935–1948, 10.1056/NEJMoa2306434. 37937763

[72] M. Fu , J. Zhao , L. Zhang , et al., “Overcoming Tyrosine Kinase Inhibitor Resistance in Lung Cancer Brain Metastasis With CTLA4 Blockade,” Cancer Cell 42, no. 11 (2024): 1882–1897.e7. 39423817 10.1016/j.ccell.2024.09.012

[73] H. Duan , J. Ren , S. Wei , et al., “Integrated Analyses of Multi‐Omic Data Derived From Paired Primary Lung Cancer and Brain Metastasis Reveal the Metabolic Vulnerability as a Novel Therapeutic Target,” Genome Medicine 16, no. 1 (2024): 138. 39593114 10.1186/s13073-024-01410-8 PMC11590298

[74] S. J. Adua , A. Arnal‐Estapé , M. Zhao , et al., “Brain Metastatic Outgrowth and Osimertinib Resistance Are Potentiated by RhoA in EGFR‐Mutant Lung Cancer,” Nature Communications 13, no. 1 (2022): 7690. 10.1038/s41467-022-34889-z PMC9744876 36509758

[75] M. Tang , M. Xu , J. Wang , et al., “Brain Metastasis From EGFR‐Mutated Non‐Small Cell Lung Cancer: Secretion of IL11 From Astrocytes Up‐Regulates PDL1 and Promotes Immune Escape,” Advanced Science 11, no. 26 (2024): e2306348. 38696655 10.1002/advs.202306348 PMC11234401

[76] H. Ruan , Z. Wang , Z. Sun , et al., “Single‐Cell RNA Sequencing Reveals the Characteristics of Cerebrospinal Fluid Tumour Environment in Breast Cancer and Lung Cancer Leptomeningeal Metastases,” Clinical and Translational Medicine 12, no. 6 (2022): e885. 35678121 10.1002/ctm2.885 PMC9178395

[77] Y. S. Li , W. P. Lai , K. Yin , et al., “Lipid‐Associated Macrophages for Osimertinib Resistance and Leptomeningeal Metastases in NSCLC,” Cell Reports 43, no. 8 (2024): 114613. 39116206 10.1016/j.celrep.2024.114613

[78] S. Couraud , F. Vaca‐Paniagua , S. Villar , et al., “Noninvasive Diagnosis of Actionable Mutations by Deep Sequencing of Circulating Free DNA in Lung Cancer From Never‐Smokers: A Proof‐Of‐Concept Study From BioCAST/IFCT‐1002,” Clinical Cancer Research 20, no. 17 (2014): 4613–4624. 25013125 10.1158/1078-0432.CCR-13-3063

[79] C. E. Blanchard , A. T. Gomeiz , K. Avery , et al., “Signaling Dynamics in Coexisting Monoclonal Cell Subpopulations Unveil Mechanisms of Resistance to Anti‐Cancer Compounds,” Cell Communication and Signaling: CCS 22, no. 1 (2024): 377. 39061010 10.1186/s12964-024-01742-3 PMC11282632

[80] C. Chen , M. P. Douglas , M. V. Ragavan , K. A. Phillips , and J. P. Jansen , “Clinical Validity and Utility of Circulating Tumor DNA (ctDNA) Testing in Advanced Non‐Small Cell Lung Cancer (aNSCLC): A Systematic Literature Review and Meta‐Analysis,” Molecular Diagnosis & Therapy 28, no. 5 (2024): 525–536. 39093546 10.1007/s40291-024-00725-x PMC11349784

[81] H. N. Nguyen , N. T. Cao , T. C. Van Nguyen , et al., “Liquid Biopsy Uncovers Distinct Patterns of DNA Methylation and Copy Number Changes in NSCLC Patients With Different EGFR‐TKI Resistant Mutations,” Scientific Reports 11, no. 1 (2021): 16436. 34385540 10.1038/s41598-021-95985-6 PMC8361064

[82] T. El Zarif , C. B. Meador , X. Qiu , et al., “Detecting Small Cell Transformation in Patients With Advanced EGFR Mutant Lung Adenocarcinoma Through Epigenomic cfDNA Profiling,” Clinical Cancer Research 30, no. 17 (2024): 3798–3811. 38912901 10.1158/1078-0432.CCR-24-0466 PMC11369616

[83] J. Song , M. H. Cho , H. Cho , et al., “Amplifying Mutational Profiling of Extracellular Vesicle mRNA With SCOPE,” Nature Biotechnology (2024): 1–11, 10.1038/s41587-024-02426-6. PMC12747296 39375445

[84] C. Lailler , A. Didelot , S. Garinet , et al., “PrP(C) Controls Epithelial‐To‐Mesenchymal Transition in EGFR‐Mutated NSCLC: Implications for TKI Resistance and Patient Follow‐Up,” Oncogene 43, no. 37 (2024): 2781–2794. 39147880 10.1038/s41388-024-03130-0 PMC11379626

[85] T. F. Hsiao , C. L. Wang , Y. C. Wu , et al., “Integrative Omics Analysis Reveals Soluble Cadherin‐3 as a Survival Predictor and an Early Monitoring Marker of EGFR Tyrosine Kinase Inhibitor Therapy in Lung Cancer,” Clinical Cancer Research 26, no. 13 (2020): 3220–3229. 32156745 10.1158/1078-0432.CCR-19-3972

[86] N. I. Vokes , E. Chambers , T. Nguyen , et al., “Concurrent TP53 Mutations Facilitate Resistance Evolution in EGFR‐Mutant Lung Adenocarcinoma,” Journal of Thoracic Oncology 17, no. 6 (2022): 779–792. 35331964 10.1016/j.jtho.2022.02.011 PMC10478031

[87] H. Isozaki , R. Sakhtemani , A. Abbasi , et al., “Therapy‐Induced APOBEC3A Drives Evolution of Persistent Cancer Cells,” Nature 620, no. 7973 (2023): 393–401. 37407818 10.1038/s41586-023-06303-1 PMC10804446

[88] K. Bai , X. Chen , X. Qi , et al., “Cerebrospinal Fluid Circulating Tumour DNA Genotyping and Survival Analysis in Lung Adenocarcinoma With Leptomeningeal Metastases,” Journal of Neuro‐Oncology 165, no. 1 (2023): 149–160. 37897649 10.1007/s11060-023-04471-8 PMC10638181

[89] Y. Wei , B. Jiang , S. Liu , et al., “Afatinib as a Potential Therapeutic Option for Patients With NSCLC With EGFR G724S,” JTO Clinical Research Reports 2, no. 7 (2021): 100193. 34590038 10.1016/j.jtocrr.2021.100193 PMC8474270

[90] G. Zhang , B. Yan , Y. Guo , H. Yang , X. Li , and J. Li , “Case Report: A Patient With the Rare Third‐Generation TKI‐Resistant Mutation EGFR L718Q Who Responded to Afatinib Plus Cetuximab Combination Therapy,” Frontiers in Oncology 12 (2022): 995624. 36387265 10.3389/fonc.2022.995624 PMC9659857

[91] Y. Wang , R. Han , M. Zhu , T. He , and Y. He , “Case Report: Durable Response to the Combination of Brigatinib and Cetuximab Plus Icotinib in a NSCLC Patient Harboring EGFR L858R‐T790M‐Cis‐G796S and L718Q Resistance Mutations Following Progression With Osimertinib,” Frontiers in Oncology 12 (2022): 875313. 35530305 10.3389/fonc.2022.875313 PMC9071300

[92] W. Fang , J. Gan , Y. Huang , H. Zhou , and L. Zhang , “Acquired EGFR L718V Mutation and Loss of T790M‐Mediated Resistance to Osimertinib in a Patient With NSCLC Who Responded to Afatinib,” Journal of Thoracic Oncology 14, no. 12 (2019): e274–e275. 31757379 10.1016/j.jtho.2019.07.018

[93] L. Lin , Q. Lu , R. Cao , et al., “Acquired Rare Recurrent EGFR Mutations as Mechanisms of Resistance to Osimertinib in Lung Cancer and In Silico Structural Modelling,” American Journal of Cancer Research 10, no. 11 (2020): 4005–4015. 33294282 PMC7716156

[94] Z. Zhou , Y. Zhao , S. Shen , et al., “Durable Clinical Response of Lung Adenocarcinoma Harboring EGFR 19Del/T790M/in Trans‐C797S to Combination Therapy of First‐ and Third‐Generation EGFR Tyrosine Kinase Inhibitors,” Journal of Thoracic Oncology 14, no. 8 (2019): e157–e159. 31075545 10.1016/j.jtho.2019.04.020

[95] Y. Wang , N. Yang , Y. Zhang , et al., “Effective Treatment of Lung Adenocarcinoma Harboring EGFR‐Activating Mutation, T790M, and Cis‐C797S Triple Mutations by Brigatinib and Cetuximab Combination Therapy,” Journal of Thoracic Oncology 15, no. 8 (2020): 1369–1375. 32353596 10.1016/j.jtho.2020.04.014

[96] Y. Yang , H. Xu , L. Ma , et al., “Possibility of Brigatinib‐Based Therapy, or Chemotherapy Plus Anti‐Angiogenic Treatment After Resistance of Osimertinib Harboring EGFR T790M‐Cis‐C797S Mutations in Lung Adenocarcinoma Patients,” Cancer Medicine 10, no. 23 (2021): 8328–8337. 34612594 10.1002/cam4.4336 PMC8633234

[97] R. Zhou , L. Song , W. Zhang , L. Shao , X. Li , and X. Li , “Combination of Osimertinib and Anlotinib May Overcome the Resistance Mediated by in Cis EGFR T790M‐C797S in NSCLC: A Case Report,” Oncotargets and Therapy 14 (2021): 2847–2851, 10.2147/OTT.S298655. 33958875 PMC8093742

[98] A. J. Cooper , L. V. Sequist , and J. J. Lin , “Third‐Generation EGFR and ALK Inhibitors: Mechanisms of Resistance and Management,” Nature Reviews. Clinical Oncology 19, no. 8 (2022): 499–514. 10.1038/s41571-022-00639-9 PMC9621058 35534623

[99] S. Wang , Y. Song , and D. Liu , “EAI045: The Fourth‐Generation EGFR Inhibitor Overcoming T790M and C797S Resistance,” Cancer Letters 385 (2017): 51–54. 27840244 10.1016/j.canlet.2016.11.008

[100] Y. Jia , C.‐H. Yun , E. Park , et al., “Overcoming EGFR(T790M) and EGFR(C797S) Resistance With Mutant‐Selective Allosteric Inhibitors,” Nature 534, no. 7605 (2016): 129–132. 27251290 10.1038/nature17960 PMC4929832

[101] S. M. Lim , T. Fujino , C. Kim , et al., “BBT‐176, a Novel Fourth‐Generation Tyrosine Kinase Inhibitor for Osimertinib‐Resistant EGFR Mutations in Non‐Small Cell Lung Cancer,” Clinical Cancer Research 29, no. 16 (2023): 3004–3016. 37249619 10.1158/1078-0432.CCR-22-3901 PMC10425724

[102] C. To , T. S. Beyett , J. Jang , et al., “An Allosteric Inhibitor Against the Therapy‐Resistant Mutant Forms of EGFR in Non‐Small Cell Lung Cancer,” Nature Cancer 3, no. 4 (2022): 402–417, 10.1038/s43018-022-00351-8. 35422503 PMC9248923

[103] Y. J. Choi , D.‐S. Kim , Y. H. Sung , et al., “The Reversible Fourth‐Generation EGFR Tyrosine Kinase Inhibitor OBX02–011 Overcomes C797S‐Mediated Resistance in Lung Cancer,” Cancer Research (2022): 1538–7445, 10.1158/0008-5472.CAN-22-0394. 35700239

[104] E. J. Lee , S. Y. Oh , Y. W. Lee , et al., “Discovery of a Novel Potent EGFR Inhibitor Against EGFR Activating Mutations and On‐Target Resistance in NSCLC,” Clinical Cancer Research 30, no. 8 (2024): 1582–1594. 38330145 10.1158/1078-0432.CCR-23-2951

[105] K. Kashima , H. Kawauchi , H. Tanimura , et al., “CH7233163 Overcomes Osimertinib‐Resistant EGFR‐Del19/T790M/C797S Mutation,” Molecular Cancer Therapeutics 19, no. 11 (2020): 2288–2297. 32943545 10.1158/1535-7163.MCT-20-0229

[106] C. To , J. Jang , T. Chen , et al., “Single and Dual Targeting of Mutant EGFR With an Allosteric Inhibitor,” Cancer Discovery 9, no. 7 (2019): 926–943, 10.1158/2159-8290.CD-18-0903. 31092401 PMC6664433

[107] S. M. Lim , S. S. Schalm , E. J. Lee , et al., “BLU‐945, a Potent and Selective Next‐Generation EGFR TKI, has Antitumor Activity in Models of Osimertinib‐Resistant Non‐Small‐Cell Lung Cancer,” Therapeutic Advances in Medical Oncology 16 (2024): 17588359241280689. 39444426 10.1177/17588359241280689 PMC11497503

[108] D. Chirnomas , K. R. Hornberger , and C. M. Crews , “Protein Degraders Enter the Clinic—A New Approach to Cancer Therapy,” Nature Reviews. Clinical Oncology 20, no. 4 (2023): 265–278. 10.1038/s41571-023-00736-3 PMC11698446 36781982

[109] Y. Du , Y. Chen , Y. Wang , et al., “HJM‐561, a Potent, Selective, and Orally Bioavailable EGFR PROTAC That Overcomes Osimertinib‐Resistant EGFR Triple Mutations,” Molecular Cancer Therapeutics 21, no. 7 (2022): 1060–1066. 35499406 10.1158/1535-7163.MCT-21-0835

[110] Y. Zhu , X. Ye , Y. Wu , et al., “Design, Synthesis, and Biological Evaluation of Novel EGFR PROTACs Targeting C797S Mutation,” Journal of Medicinal Chemistry 67, no. 9 (2024): 7283–7300. 38676656 10.1021/acs.jmedchem.4c00107

[111] E. M. Urbanska , M. Grauslund , P. R. Koffeldt , et al., “Real‐World Data on Combined EGFR‐TKI and Crizotinib Treatment for Acquired and De Novo MET Amplification in Patients With Metastatic EGFR‐Mutated NSCLC,” International Journal of Molecular Sciences 24, no. 17 (2023): 13077. 37685884 10.3390/ijms241713077 PMC10487649

[112] Y. Wang , P. Tian , L. Xia , et al., “The Clinical Efficacy of Combinatorial Therapy of EGFR‐TKI and Crizotinib in Overcoming MET Amplification‐Mediated Resistance From Prior EGFR‐TKI Therapy,” Lung Cancer 146 (2020): 165–173. 32540560 10.1016/j.lungcan.2020.06.003

[113] R. J. Hartmaier , A. A. Markovets , M. J. Ahn , et al., “Osimertinib + Savolitinib to Overcome Acquired MET‐Mediated Resistance in Epidermal Growth Factor Receptor‐Mutated, MET‐Amplified Non‐Small Cell Lung Cancer: TATTON,” Cancer Discovery 13, no. 1 (2023): 98–113. 36264123 10.1158/2159-8290.CD-22-0586 PMC9827108

[114] B. C. Cho , D. W. Kim , A. I. Spira , et al., “Amivantamab Plus Lazertinib in Osimertinib‐Relapsed EGFR‐Mutant Advanced Non‐Small Cell Lung Cancer: A Phase 1 Trial,” Nature Medicine 29, no. 10 (2023): 2577–2585. 10.1038/s41591-023-02554-7 PMC10579096 37710001

[115] J. Neijssen , R. M. F. Cardoso , K. M. Chevalier , et al., “Discovery of Amivantamab (JNJ‐61186372), a Bispecific Antibody Targeting EGFR and MET,” Journal of Biological Chemistry 296 (2021): 100641. 33839159 10.1016/j.jbc.2021.100641 PMC8113745

[116] C. K. Liam , A. R. Ahmad , T. C. Hsia , et al., “Randomized Trial of Tepotinib Plus Gefitinib Versus Chemotherapy in EGFR‐Mutant NSCLC With EGFR Inhibitor Resistance due to MET Amplification: INSIGHT Final Analysis,” Clinical Cancer Research 29, no. 10 (2023): 1879–1886. 36971777 10.1158/1078-0432.CCR-22-3318 PMC10183805

[117] Y. L. Wu , V. Guarneri , P. J. Voon , et al., “Tepotinib Plus Osimertinib in Patients With EGFR‐Mutated Non‐Small‐Cell Lung Cancer With MET Amplification Following Progression on First‐Line Osimertinib (INSIGHT 2): A Multicentre, Open‐Label, Phase 2 Trial,” Lancet Oncology 25, no. 8 (2024): 989–1002. 39089305 10.1016/S1470-2045(24)00270-5

[118] J. Rotow , J. D. Patel , M. P. Hanley , et al., “Osimertinib and Selpercatinib Efficacy, Safety, and Resistance in a Multicenter, Prospectively Treated Cohort of EGFR‐Mutant and RET Fusion‐Positive Lung Cancers,” Clinical Cancer Research 29, no. 16 (2023): 2979–2987. 36996322 10.1158/1078-0432.CCR-22-2189 PMC10524391

[119] W. Kian , B. Krayim , H. Alsana , et al., “Overcoming CEP85L‐ROS1, MKRN1‐BRAF and MET Amplification as Rare, Acquired Resistance Mutations to Osimertinib,” Frontiers in Oncology 13 (2023): 1124949. 36923435 10.3389/fonc.2023.1124949 PMC10009227

[120] Y. Li , H. Zeng , C. Qi , et al., “Features and Efficacy of Triple‐Targeted Therapy for Patients With EGFR‐Mutant Non‐Small‐Cell Lung Cancer With Acquired BRAF Alterations Who Are Resistant to Epidermal Growth Factor Receptor Tyrosine Kinase Inhibitors,” ESMO Open 9, no. 10 (2024): 103935. 39389004 10.1016/j.esmoop.2024.103935 PMC11490925

[121] Y. Zeng , Q. Zeng , B. Yang , and Y. Hu , “Therapeutic Strategies to Overcome ALK‐Fusion and BRAF‐Mutation as Acquired Resistance Mechanism in EGFR‐Mutated Non‐Small Cell Lung Cancer: Two Case Reports,” Frontiers in Oncology 14 (2024): 1390523. 39555453 10.3389/fonc.2024.1390523 PMC11563980

[122] Z. Wu , Z. Zhang , D. Zhang , and Z. Li , “Remarkable Response to Third‐Generation EGFR‐TKI Plus Crizotinib in a Patient With Pulmonary Adenocarcinoma Harboring EGFR and ROS1 Co‐Mutation: A Case Report,” Frontiers in Oncology 14 (2024): 1357230. 38476366 10.3389/fonc.2024.1357230 PMC10927992

[123] R. C. Doebele , A. Drilon , L. Paz‐Ares , et al., “Entrectinib in Patients With Advanced or Metastatic NTRK Fusion‐Positive Solid Tumours: Integrated Analysis of Three Phase 1‐2 Trials,” Lancet Oncology 21, no. 2 (2020): 271–282. 31838007 10.1016/S1470-2045(19)30691-6 PMC7461630

[124] A. Simoni‐Nieves , M. Lindzen , S. Giri , et al., “A Bispecific Antibody Targeting EGFR and AXL Delays Resistance to Osimertinib,” Cell Reports Medicine 5, no. 9 (2024): 101703. 39216477 10.1016/j.xcrm.2024.101703 PMC11528239

[125] R. Nakamura , T. Yamada , S. Tokuda , et al., “Triple Combination Therapy Comprising Osimertinib, an AXL Inhibitor, and an FGFR Inhibitor Improves the Efficacy of EGFR‐Mutated Non‐Small Cell Lung Cancer,” Cancer Letters 598 (2024): 217124. 39059573 10.1016/j.canlet.2024.217124

[126] H. Wang , Y. Liang , T. Zhang , et al., “C‐IGF1R Encoded by cIGF1R Acts as a Molecular Switch to Restrict Mitophagy of Drug‐Tolerant Persister Tumour Cells in Non‐Small Cell Lung Cancer,” Cell Death and Differentiation 30, no. 11 (2023): 2365–2381. 37689814 10.1038/s41418-023-01222-0 PMC10657401

[127] P. K. Gadekar , G. Urunkar , A. Roychowdhury , et al., “Design, Synthesis and Biological Evaluation of 2,3‐Dihydroimidazo[2,1‐b]Thiazoles as Dual EGFR and IGF1R Inhibitors,” Bioorganic Chemistry 115 (2021): 105151. 34333424 10.1016/j.bioorg.2021.105151

[128] X. Zhang , T. K. Maity , K. E. Ross , et al., “Alterations in the Global Proteome and Phosphoproteome in Third Generation EGFR TKI Resistance Reveal Drug Targets to Circumvent Resistance,” Cancer Research 81, no. 11 (2021): 3051–3066. 33727228 10.1158/0008-5472.CAN-20-2435 PMC8182571

[129] K. Fukuda , S. Otani , S. Takeuchi , et al., “Trametinib Overcomes KRAS‐G12V‐Induced Osimertinib Resistance in a Leptomeningeal Carcinomatosis Model of EGFR‐Mutant Lung Cancer,” Cancer Science 112, no. 9 (2021): 3784–3795. 34145930 10.1111/cas.15035 PMC8409422

[130] Y. Qian , S. Zhou , J. Li , et al., “Discovery of 4‐((3,4‐Dichlorophenyl)Amino)‐2‐Methylquinolin‐6‐ol Derivatives as EGFR and HDAC Dual Inhibitors,” European Journal of Pharmacology 960 (2023): 176114. 37863412 10.1016/j.ejphar.2023.176114

[131] Q. Su , E. Banks , G. Bebernitz , et al., “Discovery of (2R)‐N‐[3‐[2‐[(3‐Methoxy‐1‐Methyl‐Pyrazol‐4‐yl)Amino]Pyrimidin‐4‐yl]‐1H‐Indol‐7‐yl]‐2‐(4‐Methylpiperazin‐1‐yl)Propenamide (AZD4205) as a Potent and Selective Janus Kinase 1 Inhibitor,” Journal of Medicinal Chemistry 63, no. 9 (2020): 4517–4527. 32297743 10.1021/acs.jmedchem.9b01392

[132] X. Le , M. Nilsson , J. Goldman , et al., “Dual EGFR‐VEGF Pathway Inhibition: A Promising Strategy for Patients With EGFR‐Mutant NSCLC,” Journal of Thoracic Oncology 16, no. 2 (2021): 205–215. 33096270 10.1016/j.jtho.2020.10.006

[133] X. Le , M. B. Nilsson , J. P. Robichaux , and J. V. Heymach , “ARTEMIS Highlights VEGF Inhibitors as Effective Partners for EGFR TKIs in EGFR Mutant NSCLC,” Cancer Cell 39, no. 9 (2021): 1178–1180. 34388379 10.1016/j.ccell.2021.07.017

[134] H. Kenmotsu , K. Wakuda , K. Mori , et al., “Randomized Phase 2 Study of Osimertinib Plus Bevacizumab Versus Osimertinib for Untreated Patients With Nonsquamous NSCLC Harboring EGFR Mutations: WJOG9717L Study,” Journal of Thoracic Oncology 17, no. 9 (2022): 1098–1108. 35636696 10.1016/j.jtho.2022.05.006

[135] R. A. Soo , J. Y. Han , U. Dafni , et al., “A Randomised Phase II Study of Osimertinib and Bevacizumab Versus Osimertinib Alone as Second‐Line Targeted Treatment in Advanced NSCLC With Confirmed EGFR and Acquired T790M Mutations: The European Thoracic Oncology Platform (ETOP 10‐16) BOOSTER Trial,” Annals of Oncology 33, no. 2 (2022): 181–192. 34839016 10.1016/j.annonc.2021.11.010

[136] H. J. Chen , H. Y. Tu , Y. Hu , et al., “A Phase II Trial of Anlotinib Plus EGFR‐TKI in Advanced Non‐Small Cell Lung Cancer With Gradual, Oligo, or Potential Progression After EGFR‐TKI Treatment (CTONG‐1803/ALTER‐L001),” Journal of Hematology & Oncology 18, no. 1 (2025): 3, 10.1186/s13045-024-01656-0. 39757186 PMC11702043

[137] S. La Monica , C. Fumarola , D. Cretella , et al., “Efficacy of the CDK4/6 Dual Inhibitor Abemaciclib in EGFR‐Mutated NSCLC Cell Lines With Different Resistance Mechanisms to Osimertinib,” Cancers 13, no. 1 (2020): 6. 33374971 10.3390/cancers13010006 PMC7792603

[138] V. D. de Jager , J. A. Stigt , M. Niemantsverdriet , A. Ter Elst , and A. J. van der Wekken , “Osimertinib and Palbociclib in an EGFR‐Mutated NSCLC With Primary CDK4 Amplification After Progression Under Osimertinib,” NPJ Precision Oncology 8, no. 1 (2024): 113. 38778166 10.1038/s41698-024-00607-9 PMC11111758

[139] N. Hara , E. Ichihara , H. Kano , et al., “CDK4/6 Signaling Attenuates the Effect of Epidermal Growth Factor Receptor (EGFR) Tyrosine Kinase Inhibitors in EGFR‐Mutant Non‐Small Cell Lung Cancer,” Translational Lung Cancer Research 12, no. 10 (2023): 2098–2112. 38025818 10.21037/tlcr-23-99 PMC10654429

[140] M. N. White , Z. Piotrowska , K. Stirling , et al., “Combining Osimertinib With Chemotherapy in EGFR‐Mutant NSCLC at Progression,” Clinical Lung Cancer 22, no. 3 (2021): 201–209. 33610453 10.1016/j.cllc.2021.01.010 PMC8205932

[141] S. P. L. Saw , Y. F. Low , G. G. Y. Lai , et al., “Real‐World Outcomes of Pemetrexed‐Platinum Chemotherapy Plus Osimertinib After Progression on First‐Line Osimertinib in Advanced EGFR‐Mutated NSCLC,” Lung Cancer 193 (2024): 107856. 38889498 10.1016/j.lungcan.2024.107856

[142] A. T. M. Lee and M. Nagasaka , “CheckMate‐722: The Rise and Fall of Nivolumab With Chemotherapy in TKI‐Refractory EGFR‐Mutant NSCLC,” Lung Cancer 14 (2023): 41–46, 10.2147/LCTT.S408886. 37138950 PMC10150033

[143] J. C. Yang , D. H. Lee , J. S. Lee , et al., “Phase III KEYNOTE‐789 Study of Pemetrexed and Platinum With or Without Pembrolizumab for Tyrosine Kinase Inhibitor–Resistant, EGFR‐Mutant, Metastatic Nonsquamous Non‐Small Cell Lung Cancer,” Journal of Clinical Oncology 42, no. 34 (2024): 4029–4039, 10.1200/JCO.23.02747. 39173098 PMC11608596

[144] S. Lu , L. Wu , H. Jian , et al., “Sintilimab Plus Chemotherapy for Patients With EGFR‐Mutated Non‐Squamous Non‐Small‐Cell Lung Cancer With Disease Progression After EGFR Tyrosine‐Kinase Inhibitor Therapy (ORIENT‐31): Second Interim Analysis From a Double‐Blind, Randomised, Placebo‐Controlled, Phase 3 Trial,” Lancet Respiratory Medicine 11, no. 7 (2023): 624–636. 37156249 10.1016/S2213-2600(23)00135-2

[145] W. Fang , Y. Zhao , Y. Luo , et al., “Ivonescimab Plus Chemotherapy in Non‐Small Cell Lung Cancer With EGFR Variant: A Randomized Clinical Trial,” JAMA 332, no. 7 (2024): 561–570, 10.1001/jama.2024.10613. 38820549 PMC11337070

[146] A. Passaro , J. Wang , Y. Wang , et al., “Amivantamab Plus Chemotherapy With and Without Lazertinib in EGFR‐Mutant Advanced NSCLC After Disease Progression on Osimertinib: Primary Results From the Phase III MARIPOSA‐2 Study,” Annals of Oncology 35, no. 1 (2024): 77–90. 37879444 10.1016/j.annonc.2023.10.117

[147] F. Moik , J. M. Riedl , and C. Ay , “Correspondence to: Amivantamab Plus Chemotherapy With and Without Lazertinib in EGFR‐Mutant Advanced NSCLC After Disease Progression on Osimertinib: Primary Results From the Phase III MARIPOSA‐2 Study,” Annals of Oncology 35, no. 3 (2024): 327. 38092622 10.1016/j.annonc.2023.11.007

[148] T. Ninomaru , A. Hata , S. Hara , and M. Komatsu , “Heterogeneity or Transformation? A Whack‐A‐Mole Case of EGFR‐Mutant Lung Adenocarcinoma and Small Cell Carcinoma: A Case Report,” Thoracic Cancer 13, no. 16 (2022): 2394–2397. 35793695 10.1111/1759-7714.14563 PMC9376156

[149] C. Y. Zhang , H. Sun , J. W. Su , et al., “A Potential Treatment Option for Transformed Small‐Cell Lung Cancer on PD‐L1 Inhibitor‐Based Combination Therapy Improved Survival,” Lung Cancer 175 (2023): 68–78. 36473332 10.1016/j.lungcan.2022.11.016

[150] J. Gan , Y. Huang , J. Liao , L. Pang , and W. Fang , “HER2 Amplification in Advanced NSCLC Patients After Progression on EGFR‐TKI and Clinical Response to EGFR‐TKI Plus Pyrotinib Combination Therapy,” Oncotargets and Therapy 14 (2021): 5297–5307, 10.2147/OTT.S335217. 34824536 PMC8609241

[151] Z. Song , D. Lv , S. Q. Chen , et al., “Pyrotinib in Patients With HER2‐Amplified Advanced Non‐Small Cell Lung Cancer: A Prospective, Multicenter, Single‐Arm Trial,” Clinical Cancer Research 28, no. 3 (2022): 461–467, 10.1158/1078-0432.CCR-21-2936. 34753778

[152] M. Acchione , H. Kwon , C. M. Jochheim , and W. M. Atkins , “Impact of Linker and Conjugation Chemistry on Antigen Binding, Fc Receptor Binding and Thermal Stability of Model Antibody‐Drug Conjugates,” MAbs 4, no. 3 (2012): 362–372. 22531451 10.4161/mabs.19449 PMC3355488

[153] S. Peters , R. Stahel , L. Bubendorf , et al., “Trastuzumab Emtansine (T‐DM1) in Patients With Previously Treated HER2‐Overexpressing Metastatic Non‐Small Cell Lung Cancer: Efficacy, Safety, and Biomarkers,” Clinical Cancer Research 25, no. 1 (2019): 64–72. 30206164 10.1158/1078-0432.CCR-18-1590

[154] B. T. Li , E. F. Smit , Y. Goto , et al., “Trastuzumab Deruxtecan in HER2‐Mutant Non‐Small‐Cell Lung Cancer,” New England Journal of Medicine 386, no. 3 (2022): 241–251. 34534430 10.1056/NEJMoa2112431 PMC9066448

[155] K. Goto , Y. Goto , T. Kubo , et al., “Trastuzumab Deruxtecan in Patients With HER2‐Mutant Metastatic Non‐Small‐Cell Lung Cancer: Primary Results From the Randomized, Phase II DESTINY‐Lung02 Trial,” Journal of Clinical Oncology 41, no. 31 (2023): 4852–4863. 37694347 10.1200/JCO.23.01361 PMC10617843

[156] C. Zanchetta , L. De Marchi , M. Macerelli , et al., “Antibody‐Drug Conjugates in Non‐Small Cell Lung Cancer: State of the Art and Future Perspectives,” International Journal of Molecular Sciences 26, no. 1 (2024): 221. 39796075 10.3390/ijms26010221 PMC11719753

[157] N. Peled , W. Kian , E. Inbar , et al., “Osimertinib in Advanced EGFR‐Mutant Lung Adenocarcinoma With Asymptomatic Brain Metastases: An Open‐Label, 3‐Arm, Phase II Pilot Study,” Neurooncological Advances 4, no. 1 (2022): vdab188. 10.1093/noajnl/vdab188 PMC8826702 35156036

[158] Y. Zhou , B. Wang , J. Qu , et al., “Survival Outcomes and Symptomatic Central Nervous System (CNS) Metastasis in EGFR‐Mutant Advanced Non‐Small Cell Lung Cancer Without Baseline CNS Metastasis: Osimertinib vs. First‐Generation EGFR Tyrosine Kinase Inhibitors,” Lung Cancer 150 (2020): 178–185. 33186860 10.1016/j.lungcan.2020.10.018

[159] P. A. Jänne , D. Planchard , K. Kobayashi , et al., “CNS Efficacy of Osimertinib With or Without Chemotherapy in Epidermal Growth Factor Receptor‐Mutated Advanced Non‐Small‐Cell Lung Cancer,” Journal of Clinical Oncology 42, no. 7 (2024): 808–820. 38042525 10.1200/JCO.23.02219 PMC10906563

[160] S. Park , M. H. Lee , M. Seong , et al., “A Phase II, Multicenter, Two Cohort Study of 160 mg Osimertinib in EGFR T790M‐Positive Non‐Small‐Cell Lung Cancer Patients With Brain Metastases or Leptomeningeal Disease Who Progressed on Prior EGFR TKI Therapy,” Annals of Oncology 31, no. 10 (2020): 1397–1404. 32634610 10.1016/j.annonc.2020.06.017

[161] C. Fan , Q. Zhao , L. Li , et al., “Efficacy and Safety of Intrathecal Pemetrexed Combined With Dexamethasone for Treating Tyrosine Kinase Inhibitor‐Failed Leptomeningeal Metastases From EGFR‐Mutant NSCLC—A Prospective, Open‐Label, Single‐Arm Phase 1/2 Clinical Trial (Unique Identifier: ChiCTR1800016615),” Journal of Thoracic Oncology 16, no. 8 (2021): 1359–1368. 33989780 10.1016/j.jtho.2021.04.018

[162] D. Rosenblum , A. Gutkin , R. Kedmi , et al., “CRISPR‐Cas9 Genome Editing Using Targeted Lipid Nanoparticles for Cancer Therapy,” Science Advances 6, no. 47 (2020): eabc9450. 33208369 10.1126/sciadv.abc9450 PMC7673804

[163] K. L. Hung , J. Luebeck , S. R. Dehkordi , et al., “Targeted Profiling of Human Extrachromosomal DNA by CRISPR‐CATCH,” Nature Genetics 54, no. 11 (2022): 1746–1754. 36253572 10.1038/s41588-022-01190-0 PMC9649439

[164] D. Cheng , K. Ge , X. Yao , et al., “Tumor‐Associated Macrophages Mediate Resistance of EGFR‐TKI in Non‐Small Cell Lung Cancer: Mechanisms and Prospects,” Frontiers in Immunology 14 (2023): 1209947, 10.3389/fimmu.2023.1209947. 37649478 PMC10463184

[165] W. Park , S. Wei , C. L. Xie , et al., “Targeting Pyruvate Dehydrogenase Kinase 1 Overcomes EGFR C797S Mutation‐Driven Osimertinib Resistance in Non‐Small Cell Lung Cancer,” Experimental & Molecular Medicine 56, no. 5 (2024): 1137–1149. 38689087 10.1038/s12276-024-01221-2 PMC11148081

[166] K. R. Zhang , Y. F. Zhang , H. M. Lei , et al., “Targeting AKR1B1 Inhibits Glutathione De Novo Synthesis to Overcome Acquired Resistance to EGFR‐Targeted Therapy in Lung Cancer,” Science Translational Medicine 13, no. 614 (2021): eabg6428. 34613810 10.1126/scitranslmed.abg6428

[167] N. X. Shen , M. Y. Luo , W. M. Gu , et al., “GSTO1 Aggravates EGFR‐TKI Resistance and Tumor Metastasis via Deglutathionylation of NPM1 in Lung Adenocarcinoma,” Oncogene 43, no. 33 (2024): 2504–2516, 10.1038/s41388-024-03096-z. 38969770

[168] Y. Zhuang , K. Liu , Q. He , X. Gu , C. Jiang , and J. Wu , “Hypoxia Signaling in Cancer: Implications for Therapeutic Interventions,” MedComm 4, no. 1 (2023): e203. 36703877 10.1002/mco2.203 PMC9870816

[169] D. R. Caswell , P. Gui , M. K. Mayekar , et al., “The Role of APOBEC3B in Lung Tumor Evolution and Targeted Cancer Therapy Resistance,” Nature Genetics 56, no. 1 (2024): 60–73. 38049664 10.1038/s41588-023-01592-8 PMC10786726

